# Collinearity and Dimensionality Reduction in Radiomics: Effect of Preprocessing Parameters in Hypertrophic Cardiomyopathy Magnetic Resonance T1 and T2 Mapping

**DOI:** 10.3390/bioengineering10010080

**Published:** 2023-01-06

**Authors:** Chiara Marzi, Daniela Marfisi, Andrea Barucci, Jacopo Del Meglio, Alessio Lilli, Claudio Vignali, Mario Mascalchi, Giancarlo Casolo, Stefano Diciotti, Antonio Claudio Traino, Carlo Tessa, Marco Giannelli

**Affiliations:** 1 Institute of Applied Physics “Nello Carrara” (IFAC), Council of National Research (CNR), Sesto Fiorentino, 50019 Florence, Italy; 2 Unit of Medical Physics, Pisa University Hospital “Azienda Ospedaliero-Universitaria Pisana”, Via Roma 67, 56126 Pisa, Italy; 3 Unit of Cardiology, Azienda USL Toscana Nord Ovest, Versilia Hospital, 55041 Lido di Camaiore, Italy; 4 Unit of Radiology, Azienda USL Toscana Nord Ovest, Versilia Hospital, 55041 Lido di Camaiore, Italy; 5 Department of Experimental and Clinical Biomedical Sciences “Mario Serio”, University of Florence, 50121 Florence, Italy; 6 Department of Electrical, Electronic, and Information Engineering “Guglielmo Marconi”, University of Bologna, 47522 Cesena, Italy; 7 Unit of Radiology, Azienda USL Toscana Nord Ovest, Apuane Hospital, 54100 Massa, Italy

**Keywords:** radiomics, cardiac magnetic resonance imaging, T1 and T2 mapping, collinearity, dimensionality reduction, spatial resampling, discretization bin width, filtering, hyperthophic cardiomyopathy

## Abstract

Radiomics and artificial intelligence have the potential to become a valuable tool in clinical applications. Frequently, radiomic analyses through machine learning methods present issues caused by high dimensionality and multicollinearity, and redundant radiomic features are usually removed based on correlation analysis. We assessed the effect of preprocessing—in terms of voxel size resampling, discretization, and filtering—on correlation-based dimensionality reduction in radiomic features from cardiac T1 and T2 maps of patients with hypertrophic cardiomyopathy. For different combinations of preprocessing parameters, we performed a dimensionality reduction of radiomic features based on either Pearson’s or Spearman’s correlation coefficient, followed by the computation of the stability index. With varying resampling voxel size and discretization bin width, for both T1 and T2 maps, Pearson’s and Spearman’s dimensionality reduction produced a slightly different percentage of remaining radiomic features, with a relatively high stability index. For different filters, the remaining features’ stability was instead relatively low. Overall, the percentage of eliminated radiomic features through correlation-based dimensionality reduction was more dependent on resampling voxel size and discretization bin width for textural features than for shape or first-order features. Notably, correlation-based dimensionality reduction was less sensitive to preprocessing when considering radiomic features from T2 compared with T1 maps.

## 1. Introduction

Radiomics is a novel tool allowing the extraction of many quantitative morphological, histogram-based, and textural characteristics (i.e., radiomic features) from digital medical images [1]. The underlying idea is that medical images are actually data containing objective and quantitative information, which is not obtainable from qualitative visual inspection as usually performed in routine clinical practice. Artificial intelligence (AI) methods applied to radiomic data have the potential to become a useful tool in a clinical setting, supporting clinical practice and, at the same time, medical understanding of diseases [2]. On the other hand, given the extraction of a large amount of features, AI methods applied for radiomic analyses present issues of high-dimensionality data [2]. In addition, while hundreds or thousands of radiomic features can be extracted, the sample sizes of datasets usually available in clinical studies are much smaller. Therefore, when the number of dimensions is larger than the number of samples, multicollinearity must be taken into account as another confounding effect in the analysis [3]. In this case, at least one of the variables can be expressed as a linear combination of the others. This kind of correlation among variables could affect the subsequent analyses and the results interpretation. Indeed, from a computational point of view, a group of highly correlated features will not bring additional information (or just very few) but will increase the complexity of the algorithm. Remembering the principle of parsimony (Occam’s razor), with no significant difference in performance, simpler models should be preferred [4]. In addition, in the field of medicine, interpretation of results is of paramount importance. Although some conventional statistical models and machine learning algorithms can appear simple and interpretable (e.g., decision trees), their results can be biased in the presence of correlated input features.

For these reasons, redundant radiomic features are usually removed based on the results of a correlation analysis. This step is critical because the right choice of which features should be eliminated could either improve the performance and explainability of the AI model or affect its performance by removing interesting correlations between features and the study objective [5]. In the previous literature, extensive examples of dimensionality reduction in radiomic features based on correlation analysis have been provided [2,6,7,8,9,10,11,12,13,14,15,16,17,18,19,20,21,22,23,24,25,26,27,28,29,30,31,32].

Image resampling and discretization, as well as filtering, are some steps of the radiomic workflow that may be performed as preprocessing before radiomic features extraction from the acquired image data [33,34]. In particular, image interpolation at the same voxel size is a common and recommended practice (especially in retrospective studies) to reduce any heterogeneity in acquisition voxel size. In contrast, image discretization is required to ensure that textural features estimation is computationally less burdensome [33,35]. Moreover, applying filters before radiomic features estimation could allow uncovering further tissue characteristics. Previous studies have investigated the dependence of radiomic features extraction on different image preprocessings for various applications, showing an appreciable (low or high) sensitivity of radiomic features estimate to preprocessing [36,37,38,39,40]. For instance, Marfisi et al. [37], investigating the effect of image preprocessing (in terms of image resampling, discretization, and filtering) on radiomic features from cardiac T1 and T2 mapping, have found a remarkable dependence of feature estimates on image filters, while the sensitivity of many radiomic features to image resampling and discretization was limited. On the other hand, in computed tomography imaging, the effect of image resampling can be clearly appreciable for first-order features and relevant for textural features [40]. Traverso et al. have shown that textural features derived from apparent diffusion coefficient maps appear to be highly or moderately sensitive to image preprocessing [41]. However, to the best of our knowledge, no work has assessed the dependence of dimensionality reduction based on collinearity analysis on image preprocessing.

Cardiac magnetic resonance (CMR) has a crucial role in diagnosis, risk stratification, and treatment planning in hypertrophic cardiomiopathy (HCM) [42], a genetically determined disease that affects about 1 in 500 people in the general adult population [43]. Traditionally, CMR evaluation of HCM patients relied on cine steady-state free precession images, utilized to define myocardial thickness and to calculate ventricular function, as well as on late gadolinium enhancement sequences for the identification of focal myocardial fibrosis, which has been demonstrated to have a negative prognostic value. Moreover, classic T2 STIR (short tau inversion recovery) sequences can be employed to detect myocardial edema, which has a role in arrythmic risk stratification.

In recent years, in addition to the traditional CMR sequences, T1 and T2 mapping sequences have been developed, allowing a more quantitative evaluation of myocardial changes, signally fibrosis (T1 maps), and edema (T1 and T2 maps). T1 and T2 mapping have shown to feature a possible important diagnostic and prognostic role in HCM patients (see [44] for a review). Radiomic analysis of T1 and T2 maps is hence particularly attractive, as it could allow overcoming some limitations of T1 and T2 values that have been demonstrated to suffer possible overlap between different myocardial diseases, as well as between patients and healthy controls [45,46,47,48,49]. For a proper clinical application of radiomics, the influence of each step of the radiomic workflow on features estimation should be considered. Notwithstanding, the effect of preprocessing on radiomic features selection is not usually considered in clinical studies.

Therefore, in the present study, we aimed to comprehensively assess the effect of preprocessing—in terms of voxel size resampling, discretization, and filtering—on correlation-based dimensionality reduction in radiomic features from quantitative cardiac T1 and T2 maps in a group of patients with HCM.

## 2. Materials and Methods

### 2.1. Dataset

Between November 2013 and July 2020, twenty-six patients with known or suspected HCM were referred for clinical cardiac magnetic resonance imaging (MRI). A complete MRI scan with both T1 and T2 mapping sequences was executed for these patients. The HCM diagnoses were made following the most recent recommendations of the European Society of Cardiology. They were based on the detection of the left ventricular wall thickness ≥15 mm in one or more myocardial segments, which was not due mainly to loading conditions [42]. Table 1 provides details about the patient group’s clinical and cardiac MRI-derived characteristics.

### 2.2. Cardiac MRI

A 1.5 T MRI scanning system (MAGNETOM Avanto, Siemens Healthcare, Erlangen, Germany) with 45 mT/m gradients strength and a 12-channel phased array coil was used for all cardiac MRI examinations.

Cine scans were obtained using a TrueFISP sequence (TR = 2.5 ms, TE = 1.2 ms, slice thickness = 8 mm) in the 2- and 4-chamber view planes (3 slices each), as well as in the short-axis view (8–14 slices comprising the entire left ventricle).

The short-axis view was used to obtain both T1 and T2 maps (a single slice located where myocardial thickness, evaluated with cine images, was maximum). A modified look-locker inversion recovery (MOLLI) pulse sequence with a 3-3-5 acquisition method was employed to perform T1 mapping [50]. The following pulse sequence parameters were used: TE/TR = 1.14/2.5 ms, flip angle = 35°, matrix size = 126 × 192, in-plane resolution ranged from 1.77 mm × 1.77 mm to 2.34 mm × 2.34 mm, typical field of view = 380 mm × 275 mm, and slice thickness = 8 mm. T2 maps were obtained using a T2-prepared TrueFISP sequence [51] with the following parameters: in-plane resolution ranged from 1.77 mm × 1.77 mm to 2.34 mm × 2.34 mm, typical field of view was 380 mm × 275 mm, slice thickness was 6 mm, T2 preparation time was 0/24/55 ms, TR was 4 R-R, flip angle was 70°, and matrix size was 126 × 192. Then, 10–15 min after intravenous gadolinium DTPA (Magnevist, Schering), gadoteric acid (Dotarem, Guerbet), or gadoteridol (Prohance, Bracco) administration, late gadolinium enhancement (LGE) images were acquired in the 2- and 4-chamber views of the left ventricle (LV) (3 slices each), as well as in the short-axis view (8–14 slices encompassing the entire left ventricle) using a 2D phase-sensitive inversion recovery (PSIR) sequence (TR = 700 ms, TE = 1.09 ms, slice thickness = 8 mm, inversion time = 200–300 ms, and typical in-plane resolution = 2.4 mm × 3.2 mm).

### 2.3. T1 and T2 Maps Preprocessing

**Figure 1:**
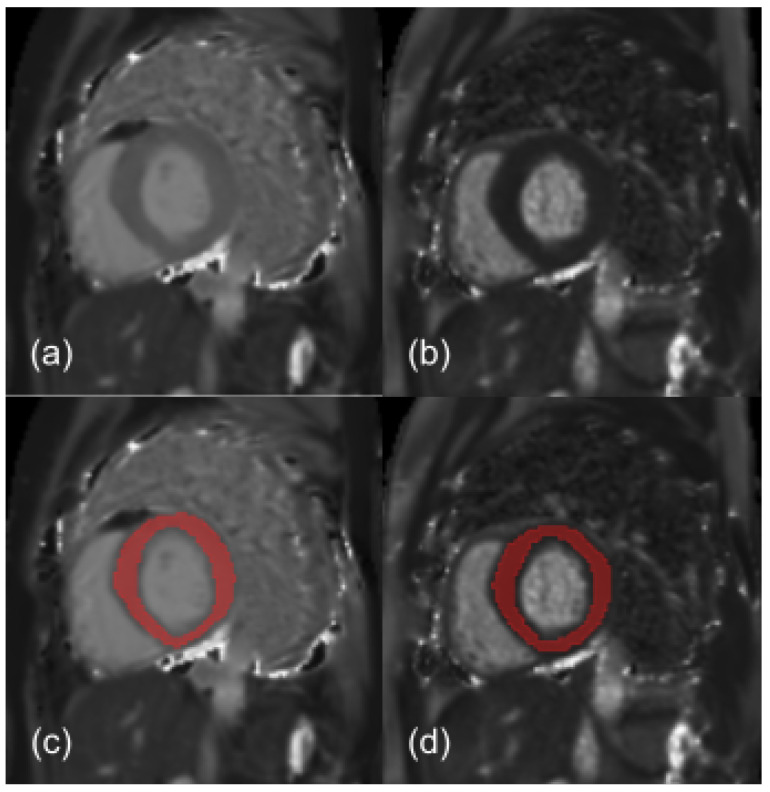
T1 (**a**) and T2 (**b**) maps (short-axis view) of a representative HCM patient, with the corresponding manually segmented myocardium region of interest (ROI) showed in panel (**c**,**d**), respectively. Myocardium area size ranged from 740 mm^2^ to 2370 mm^2^ across enrolled HCM patients. Typical myocardial T1 and T2 values were approximately 997 ms and 53 ms (median values across HCM patients), respectively.

A cardiac MRI specialist with 15 years of expertise manually segmented the whole myocardium of each subject using 3D Slicer software (version 4.11.2) [52,53]. In order to avoid partial volume effects, the myocardium area was independently defined on T1 and T2 maps. A myocardial segmentation for a representative HCM patient is shown in Figure 1.

In this study, we independently applied three preprocessing steps on T1 and T2 maps: (1) voxel size resampling, (2) discretization, and (3) filtering.

Given that T1 and T2 mapping only enabled the acquisition of a single slice, we performed voxel size resampling by 2D interpolation using the B-spline interpolation algorithm (with the origins of interpolation and original image grids aligned together [33]). Calculated T1 and T2 maps, which had an in-plane spatial resolution varying across subjects from 1.77 mm × 1.77 mm to 2.34 mm × 2.34 mm, were resampled to achieve in-plane isotropic spatial resolutions of 1.8 mm, 1.9 mm, 2.0 mm, 2.1 mm, 2.2 mm, 2.3 mm, and 2.4 mm.

The Image Biomarker Standardisation Initiative (IBSI) has suggested carrying out image discretization with fixed bin width when dealing with quantitative data, such as T1 and T2 maps [33]. In this study, for each resampling voxel size, the discretization bin width values used for T1 maps were 3.60 ms, 3.95 ms, 4.30 ms, 4.65 ms, 5.00 ms, 5.35 ms, 5.70 ms, 6.05 ms, and 6.40 ms, while bin width values used for T2 maps were 0.49 ms, 0.50 ms, 0.51 ms, 0.52 ms, 0.53 ms, 0.54 ms, 0.55 ms, 0.56 ms, and 0.57 ms. Specifically, bin width values were chosen so that the number of quantization levels for T1 and T2 maps was within the range of 30–130 for each HCM patient. This approach, previously used in other technical investigations [41,54,55,56], may reduce the variability in estimating radiomic features [57,58,59].

Different filters were applied to T1 and T2 maps, including the gradient magnitude of the map (i.e., gradient filter), the square of the map values (i.e., square filter), the square root of the absolute map value (i.e., square-root filter), and 2D wavelets (Daubechies 3). The last one yielded four filtered maps obtained through different combinations of 2D wavelets (i.e., wavelet-LH, -HL, -HH, and -LL), where L/H refers to the combination of low-/high filters applied in the horizontal and vertical direction. Specifically, filtering was carried out at fixed isotropic in-plane resampling voxel size of 2.1 mm and at fixed discretization bin width of 6 ms and 0.56 ms for T1 and T2 maps, respectively. These bin width values ensured a median (across subjects) number of quantization levels between 30 and 130 [57,58,59].

All preprocessing steps and subsequent radiomic features estimations were carried out by using the open-source PyRadiomics library [60] (version 3.0.1) and Python (version 3.7.3) running on a MacBook Air (macOS version 10.14) with a 1.8 GHz Intel Core i5 CPU.

### 2.4. Radiomic Features Estimation

Given that the used acquisition sequences allowed obtaining T1 and T2 maps on a single slice, the 2D versions of radiomic features were considered. For each preprocessing combination, in terms of resampling voxel size and discretization bin width, a total of 98 features were obtained from both T1 and T2 maps: 9 2D shape features, 16 first-order features (14 intensity-based statistical features and two intensity histogram features, namely Entropy and Uniformity), and 73 second-order features (i.e., textural features) from gray-level co-occurrence matrix (GLCM, 22 features), gray-level run length matrix (GLRLM, 16 features), gray-level size zone matrix (GLSZM, 16 features), gray-level dependence matrix (GLDM, 14 features, with coarseness parameter α=0), and neighborhood gray-tone difference matrix (NGTDM, 5 features). Second-order features estimation was performed according to the Chebyshev norm with a distance of 1 pixel. GLCM and GLRLM features were computed from each 2D directional matrix (i.e., at 0°, 45°, 90°, and 135°) and averaged over 2D directions.

For each filter applied on T1 and T2 maps, 89 features were estimated for both T1 and T2 maps, i.e., all the above except the shape features. Indeed, given that shape features are usually estimated regardless of the applied image filter, they were not included in our analysis.

All radiomic features were computed following the definitions provided by the IBSI [33]. It is worth noting that the first-order feature of Kurtosis calculated by PyRadiomics was in accordance with the IBSI, except for an offset value (i.e., 3).

### 2.5. Collinearity Analysis and Dimensionality Reduction

For T1 and T2 maps, three different effects on radiomic features collinearity and dimensionality reduction were assessed:

**Effect A**  —for each discretization bin width, the effect of using different resampling voxel sizes;
**Effect B**  —for each resampling voxel size, the effect of using different discretization bin widths;
**Effect C**  —at fixed resampling voxel size and discretization bin width, the effect of using different filters.

For all combinations of preprocessing (in terms of resampling voxel size, discretization bin width, and filter), we performed a collinearity analysis by computing the pair-wise Pearson’s correlation coefficient (PCC) [61] and the Spearman’s correlation coefficient (SCC) [62] for each couple of radiomic features’ values across subjects. All significant correlation coefficients (*p*-value < 0.05) with absolute value above a predefined threshold were counted and represented in a correlation heatmap. In particular, according to previous studies, we considered cut-off thresholds of 0.8 and 0.9 for the |PCC| [2,7,18,19,63] and the |SCC| [15,22,26,30], respectively.

Subsequently, we executed an iterative dimensionality reduction in radiomic features based on either the PCC or the SCC. For the pair of features with the highest absolute correlation coefficient, we computed the mean absolute correlation coefficient of each of the two features with all the others, removing the feature with the highest mean absolute correlation coefficient. We iteratively repeated each step until the pair-wise correlation coefficients among radiomic features became less than the predefined threshold value.

Using this procedure, from the original radiomic features data, we obtained a specific set of radiomic features with lower dimensionality and redundancy for each combination of preprocessing. Therefore, we (1) compared the number of significant correlations with an absolute correlation coefficient greater than or equal to the threshold value, (2) evaluated the percentage of remaining features after the correlation-based dimensionality reduction, and (3) analyzed the differences in the remaining feature subsets by measuring a stability index [64,65].

The stability index has been proposed by several authors for the study of feature selection, showing how even slight variations in the data can lead to different sets of selected features, in terms of both cardinality and type [64,65,66,67,68,69]. In this work, we computed a measure that belongs to stability by Index/Subset category [70,71]. Briefly, a subset of remaining features is represented as a binary vector, where 0 represents absence and 1 represents the presence of the specific feature. The stability is calculated by the amount of overlap between the overall subsets of remaining features. Specifically, we used the stability index defined by Nogueira et al., which complies with the properties of a stability measure [66]. This stability index takes continuous values between 0 (lowest stability) and 1 (highest stability). In accordance with the work by Kuncheva et al., stability is considered good if it is greater than or equal to 0.5 [64]. This index can be actually used to analyze correlation-based dimensionality reduction, helping us to understand whether preprocessing can introduce changes in the data such as to yield different sets of selected features in terms of both cardinality and type.

The collinearity analysis, dimensionality reduction, and stability analysis were carried out using in-house written Python code (Version 3.10.4) running on an M1 MacBook Air (macOS Monterey version 12.3.1). In particular, we computed the stability index using the Python package freely available at https://github.com/nogueirs/JMLR2018 (accessed on 15 January 2021) [66].

## 3. Results

### 3.1. T1 Mapping

When varying resampling voxel size and discretization bin width (i.e., effect A and B, respectively), both the PCC- and SCC-based correlation analysis showed different numbers of significant pair-wise correlation coefficients with an absolute value greater than or equal to the defined threshold values. For T1 mapping, for instance, when considering the resampling voxel sizes of [1.8, 1.9, 2.0, 2.1, 2.1, 2.3, 2.4] mm, the corresponding numbers of significant |PCC| values greater than 0.8 were [903, 774, 660, 793, 806, 778, 659] and [882, 799, 676, 800, 839, 851, 655] for fixed discretization bin width values of 3.60 ms and 3.95 ms, respectively (effect A in Table 2). For the same resampling voxel sizes, the number of significant |SCC| values greater than 0.9 were [497, 447, 507, 438, 491, 461, 392] for discretization bin width = 3.60 ms and [490, 489, 510, 416, 466, 498, 413] for discretization bin width = 3.95 ms. A similar result can be observed for all the discretization bin width values and resampling voxel sizes, as reported in Table 2 for effect A and B and in Supplementary Figures S1–S4.

Different numbers of significant pair-wise correlations between features with absolute correlation coefficient value greater than the defined threshold may yield different percentages of features remaining downstream of dimensionality reduction. With reference to the representative abovementioned example, as the resampling voxel size changed, the percentages of features remaining after the PCC-based dimensionality reduction were [21, 24, 24, 22, 23, 23, 23]% and [21, 24, 23, 23, 22, 22, 24]% for bin width = 3.60 ms and bin width = 3.95 ms, respectively. On the other hand, for the SCC-based dimensionality reduction, the percentages of remaining features were [28, 28, 29, 27, 27, 32, 31]% and [29, 29, 26, 29, 29, 31, 29]%. As shown in Table 2, for both PCC- and SCC-based dimensionality reduction, at fixed discretization bin width (effect A) and resampling voxel size (effect B), the percentage of remaining features changes only slightly when varying resampling voxel size and discretization bin width, respectively. Nonetheless, it is also important to assess whether and to what extent the type of remaining features is dependent on a specific preprocessing element.

In Table 3, the results of the stability analysis are reported in detail. For both effects A and B, the stability of the features remaining after the dimensionality reduction can be considered relatively high, given that all the stability indices were greater than 0.5 [64].

On the other hand, as the type of filtering varies, the number of significant pair-wise correlations with absolute correlation coefficient values greater than the predefined threshold can differ greatly (Supplementary Figures S5 and S6). In particular, for both PCC and SCC analysis, applying a gradient filter on the original T1 maps leads to a relevant increase in the number of significant correlation coefficients with absolute value greater than the predefined threshold (see Table 2). While the percentage of features remaining downstream of dimensionality reduction seems to vary slightly with filtering, the stability of the remaining feature subsets is relatively low (less than 0.5 [64]), confirming the sensitivity of correlation-based dimensionality reduction to the application of different filters (Table 3).

**Figure 2:**
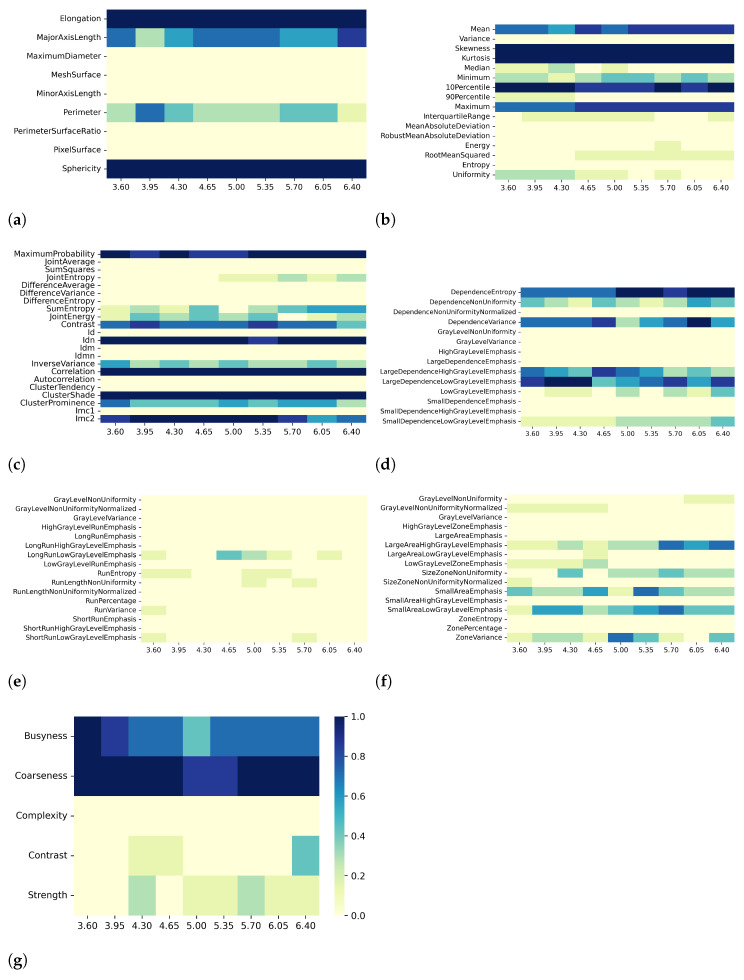
Heatmaps of remaining features from T1 maps after PCC-based dimensionality reduction for effect A (varying resampling voxel size, with fixed discretization bin width (ms)). The radiomic features are separated in different classes: (**a**) 2D shape, (**b**) first order, (**c**) GLCM, (**d**) GLDM, (**e**) GLRLM, (**f**) GLSZM, (**g**) NGTDM.

**Figure 3:**
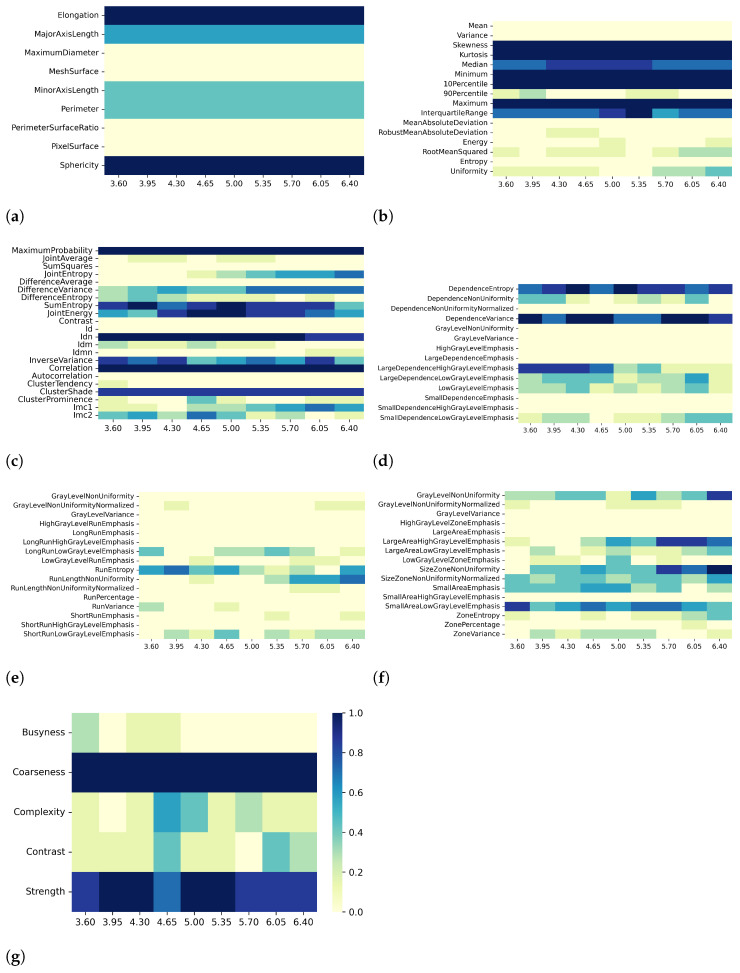
Heatmaps of remaining features from T1 maps after SCC-based dimensionality reduction for effect A (varying resampling voxel size, with fixed discretization bin width (ms)). The radiomic features are separated in classes: (**a**) 2D shape, (**b**) first order, (**c**) GLCM, (**d**) GLDM, (**e**) GLRLM, (**f**) GLSZM, (**g**) NGTDM.

**Figure 4:**
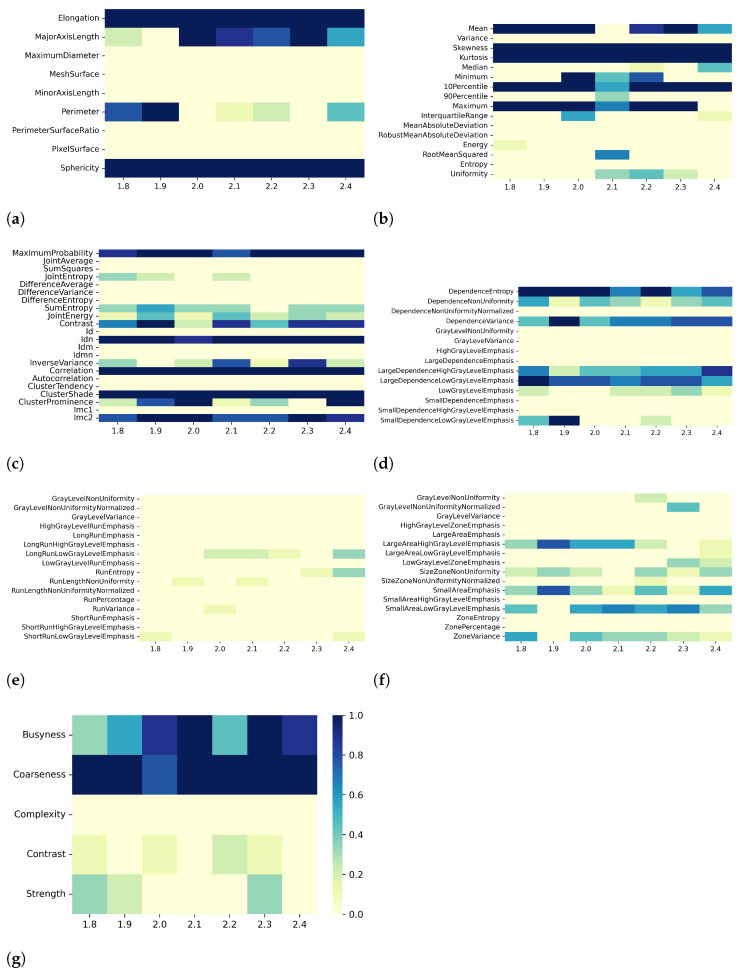
Heatmaps of remaining features from T1 maps after PCC-based dimensionality reduction for effect B (varying discretization bin width, with fixed resampling voxel size (mm)). The radiomic features are separated in classes: (**a**) 2D shape, (**b**) first order, (**c**) GLCM, (**d**) GLDM, (**e**) GLRLM, (**f**) GLSZM, (**g**) NGTDM.

**Figure 5:**
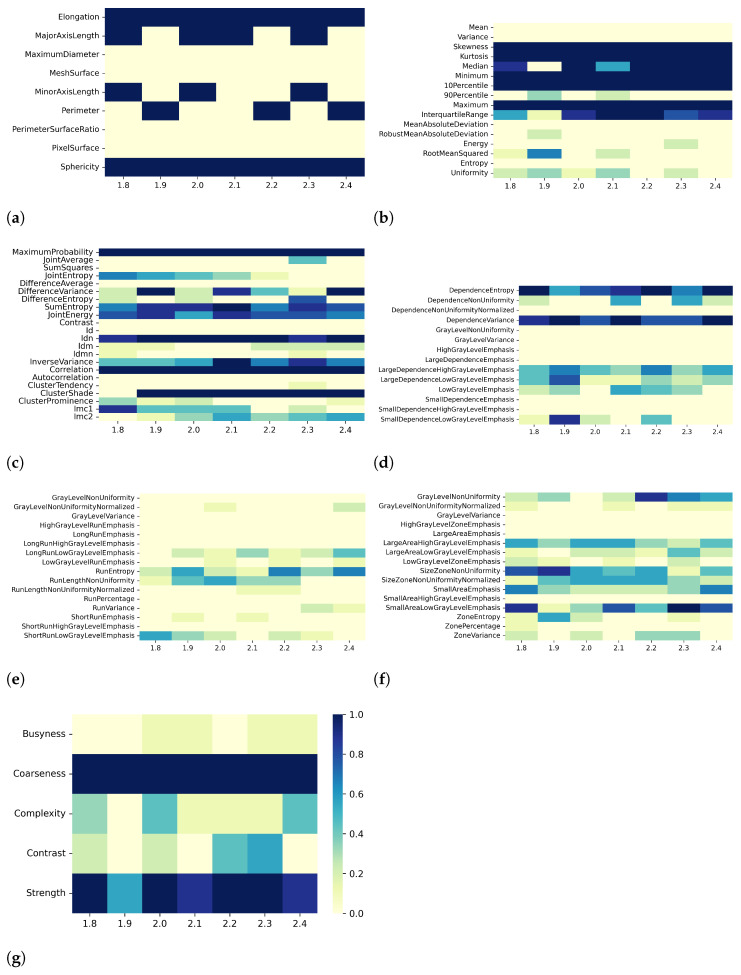
Heatmaps of remaining features from T1 maps after SCC-based dimensionality reduction for effect B (varying discretization bin width, with fixed resampling voxel size (mm)). The radiomic features are separated in classes: (**a**) 2D shape, (**b**) first order, (**c**) GLCM, (**d**) GLDM, (**e**) GLRLM, (**f**) GLSZM, (**g**) NGTDM.

Heatmaps in Figure 2, Figure 3, Figure 4 and Figure 5 show in greater detail the relationship between each preprocessing and the specific radiomic features that are retained or eliminated by correlation-based dimensionality reduction. In particular, by fixing one resampling voxel size or discretization bin width while varying the other, these heatmaps represent the ratio between the number of times each radiomic feature was selected and the total number of times the variable could be selected (i.e., the number of considered preprocessing combinations) through the dimensionality reduction process. For T1 mapping, the features belonging to the GLRLM class were almost always removed, indicating high collinearity with the other features (Figure 2e, Figure 3e, Figure 4e, and Figure 5e). For the other classes of features, only a few specific ones were always eliminated, such as: MaximumDiameter, MeshSurface, PerimeterSurfaceRatio, and PixelSurface [2D shape (Figure 2a, Figure 3a, Figure 4a and Figure 5a)], Variance, MeanAbsoluteDeviation, Entropy [first-order (Figure 2b, Figure 3b, Figure 4b and Figure 5b)], SumSquares, Id, AutoCorrelation [GLCM (Figure 2c, Figure 3c, Figure 4c, and Figure 5c)], DependenceNonUniformityNormalized, GrayLevelNonuniformity, GrayLevelVariance, HighGrayLevelEmphasis, LargeDependenceEmphasis, SmallDependenceEmphasis, SmallDependenceHighGrayLevelEmphasis [GLDM (Figure 2d, Figure 3d, Figure 4d and Figure 5d)], and GrayLevelVariance, HighGrayLevelZoneEmphasis, LargeAreaEmphasis [GLSZM (Figure 2f, Figure 3f, Figure 4f, and Figure 5f)].

### 3.2. T2 Mapping

Overall, the results for T2 mapping are similar to those for T1 maps. Specifically, when varying resampling voxel size and discretization bin width (i.e., effect A and B, respectively), both the PCC- and SCC-based correlation analysis showed different numbers of pair-wise significant correlation coefficients with absolute value greater than or equal to the defined threshold value. For instance, when considering the discretization bin width values of [0.49, 0.50, 0.51, 0.52, 0.53, 0.54, 0.55, 0.56, 0.57] ms, the number of significant correlations with |PCC| values greater than 0.8 were [553, 570, 574, 564, 558, 572, 582, 555, 583] and [579, 582, 594, 574, 582, 589, 579, 599, 591] for fixed resampling voxel sizes of 1.8 mm and 1.9 mm, respectively (effect B in Table 4). At the same values of discretization bin width, the number of significant correlations with |SCC| values greater than 0.9 were [417, 414, 403, 386, 419, 399, 415, 413, 413] for the resampling voxel size of 1.8 mm and [445, 432, 460, 431, 439, 442, 426, 467, 426] for the resampling voxel size of 1.9 mm. In Table 4 and in the correlation heatmaps in Supplementary Figures S7–S10, a similar result can be observed for all preprocessing combinations of effects A and B. Following the abovementioned example, as the discretization bin width changed, the percentages of features remaining after the PCC-based dimensionality reduction were [24, 24, 24, 23, 24, 23, 23, 24, 24]% and [23, 21, 22, 22, 26, 22, 22, 22, 21]% for resampling voxel sizes of 1.8 mm and 1.9 mm, respectively. On the other hand, for the SCC-based dimensionality reduction, the percentages of remaining features were [32, 33, 34, 34, 31, 33, 32, 31, 32]% and [32, 32, 33, 32, 32, 35, 30, 31, 34]% for resampling voxel sizes of 1.8 mm and 1.9 mm, respectively. As observed for T1 mapping, and reported in detail in Table 4, for both PCC- and SCC-based dimensionality reduction, at fixed discretization bin width (i.e., effect A) and resampling voxel size (i.e., effect B), the percentage of remaining features changes only slightly with varying resampling voxel size and discretization bin width, respectively.

Moreover, the results of the stability analysis, reported in Table 5, indicate that, regardless of the effects A and B, the stability of the features remaining after the dimensionality reduction can be considered relatively high (all the stability indices were greater than 0.5) [64].

The number of significant pair-wise correlations between features with absolute correlation coefficient value greater than the predefined threshold was greatly dependent on the applied filter (Supplementary Figures S11 and S12). In particular, for both PCC and SCC analysis, applying a gradient filter on the original maps yielded a clear increase in the number of significant correlations between features with absolute correlation coefficient value greater than the predefined threshold (see Table 4). As indicated in Table 5, the stability index of the remaining feature subsets is relatively low (less than 0.5), confirming also for T2 maps the sensitivity of correlation-based dimensionality reduction to the application of different filters.

**Figure 6:**
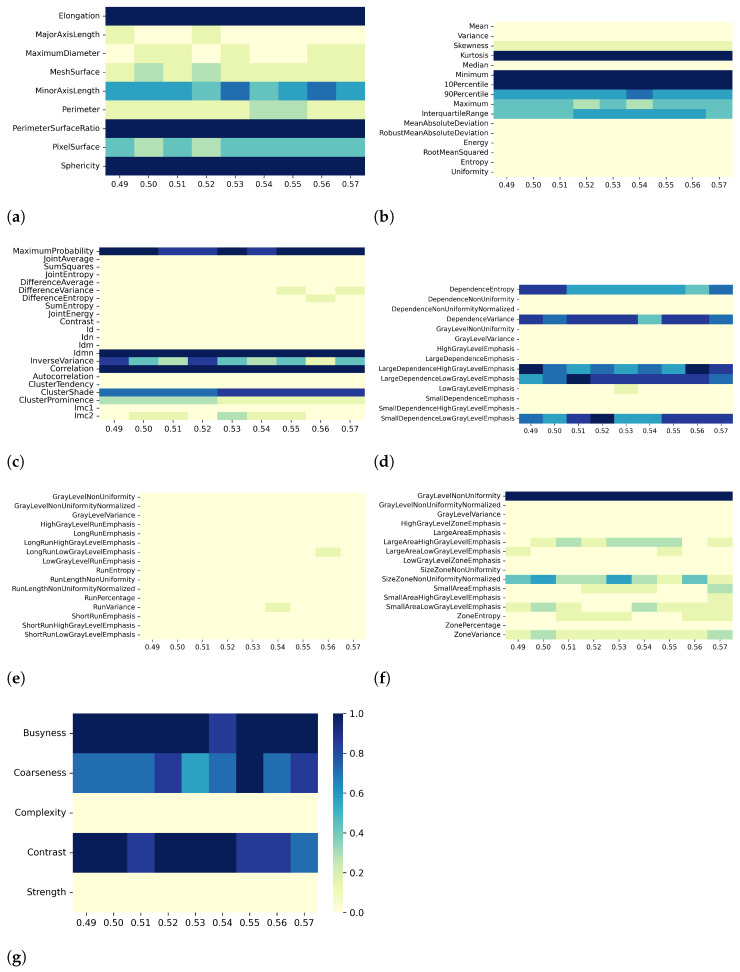
Heatmaps of remaining features from T2 maps after PCC-based dimensionality reduction for effect A (varying resampling voxel size, with fixed discretization bin width (ms)). The radiomic features are separated in classes: (**a**) 2D shape, (**b**) first order, (**c**) GLCM, (**d**) GLDM, (**e**) GLRLM, (**f**) GLSZM, (**g**) NGTDM.

**Figure 7:**
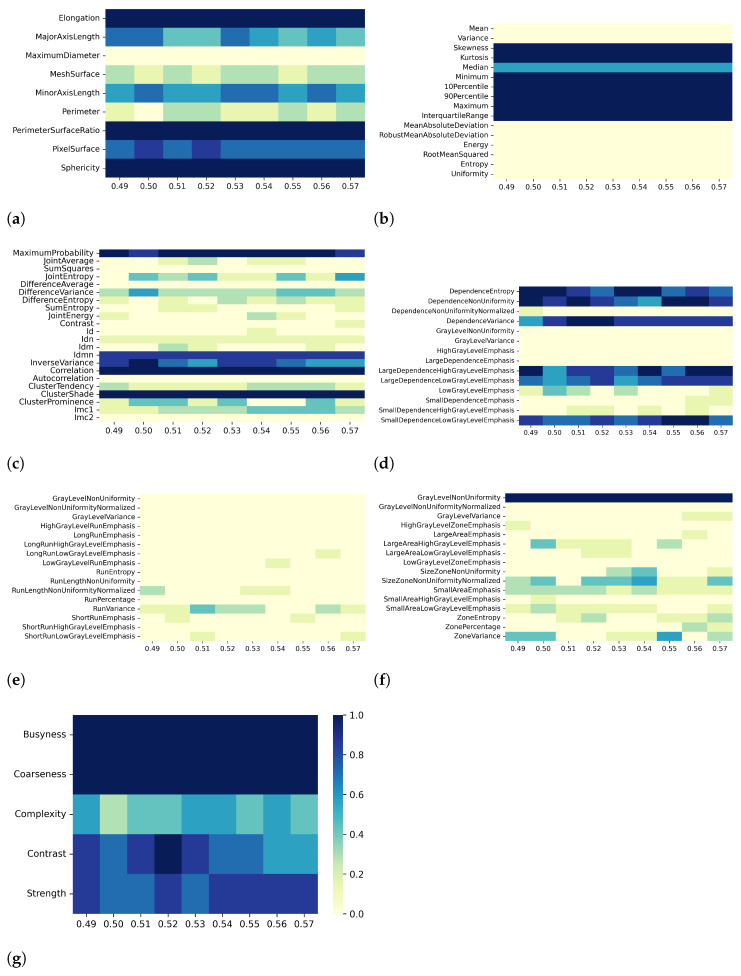
Heatmaps of remaining features from T2 maps after SCC-based dimensionality reduction for effect A (varying resampling voxel size, with fixed discretization bin width (ms)). The radiomic features are separated in classes: (**a**) 2D shape, (**b**) first order, (**c**) GLCM, (**d**) GLDM, (**e**) GLRLM, (**f**) GLSZM, (**g**) NGTDM.

**Figure 8:**
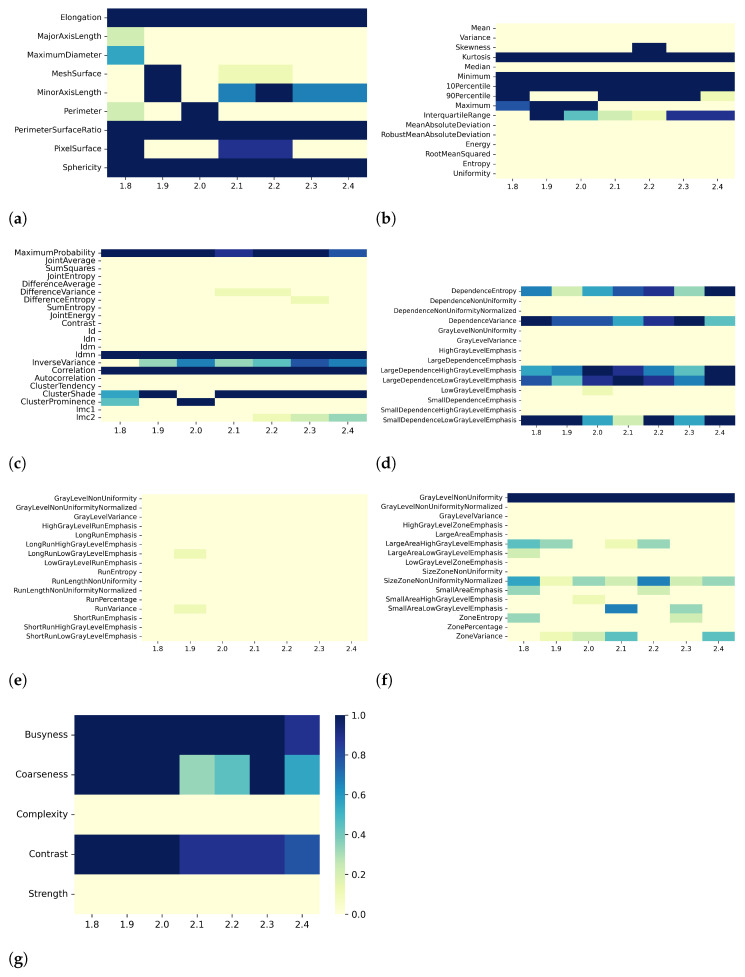
Heatmaps of remaining features from T2 maps after PCC-based dimensionality reduction for effect B (varying discretization bin width, with fixed resampling voxel size (mm)). The radiomic features are separated in classes: (**a**) 2D shape, (**b**) first order, (**c**) GLCM, (**d**) GLDM, (**e**) GLRLM, (**f**) GLSZM, (**g**) NGTDM.

**Figure 9:**
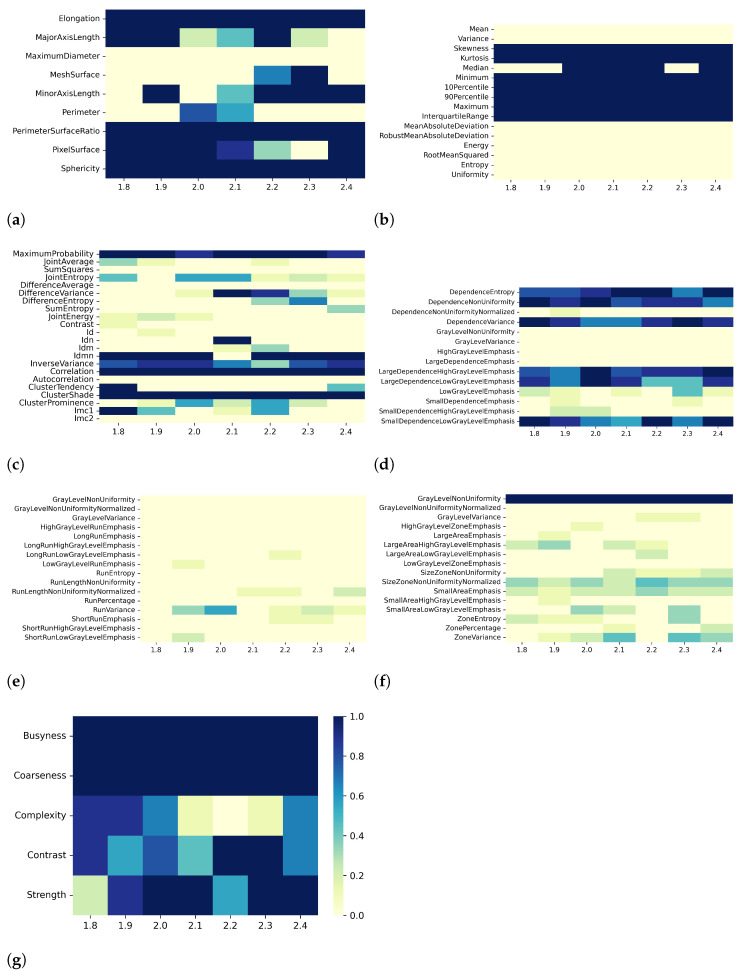
Heatmaps of remaining features from T2 maps after SCC-based dimensionality reduction for effect B (varying discretization bin width, with fixed resampling voxel size (mm)). The radiomic features are separated in classes: (**a**) 2D shape, (**b**) first order, (**c**) GLCM, (**d**) GLDM, (**e**) GLRLM, (**f**) GLSZM, (**g**) NGTDM.

Figure 6, Figure 7, Figure 8 and Figure 9 show the heatmaps of remaining features downstream of the dimensionality reduction process. The features belonging to the GLRLM category were almost always removed, indicating high collinearity with the other features (Figure 6e, Figure 7e, Figure 8e, and Figure 9e). For the other classes of features, only a few specific ones were always eliminated: Mean, Variance, MeanAbsoluteDeviation, RobustMeanAbsoluteDeviation, Energy, RooMeanSquared, Entropy, Uniformity (first-order (Figure 6b, Figure 7b, Figure 8b, and Figure 9b)), SumSquares, Autocorrelation (GLCM (Figure 6c, Figure 7c, Figure 8c, and Figure 9c)), GrayLevelNonuniformity, GrayLevelVariance, HighGrayLevelEmphasis, LargeDependenceEmphasis (GLDM (Figure 6d, Figure 7d, Figure 8d, and Figure 9d), GrayLevelNonUniformityNormalized, and LowGrayLevelZoneEmphasis (GLSZM (Figure 6f, Figure 7f, Figure 8f, and Figure 9f).

## 4. Discussion

The effect of preprocessing on radiomic features dimensionality reduction is not usually considered in clinical studies. Therefore, we assessed in detail, for the first time, whether and how voxel size resampling and discretization, as well as filtering, can impact on radiomic features selection, considering the specific case of myocardial T1 and T2 mapping-derived features of a homogenous group of patients with HCM.

For both T1 and T2 maps, the dependence of collinearity of radiomic features and correlation-based dimensionality reduction with respect to preprocessing, in terms of maps resampling and discretization, results relatively moderate. In fact, for all considered cases, the percentage of features downstream of correlation-based dimensionality reduction varies only slightly with resampling voxel size and discretization bin width, within an interval equal to [19–35]%. In addition, the stability index is always greater than 0.5, indicating that the feature sets remaining after dimensionality reduction not only have similar cardinality when applying different resampling voxel sizes or discretization bin widths but also exhibit almost the same type of radiomic features. Overall, the number of eliminated radiomic features through correlation-based dimensionality reduction is more dependent on resampling voxel size and discretization bin width for textural features than for shape or first-order features. Figure 2, Figure 3, Figure 4, Figure 5, Figure 6, Figure 7, Figure 8 and Figure 9 clearly show that the textural features remaining after correlation-based dimensionality reduction (panels c–g) vary with preprocessing changes more than the shape (panel a) or first-order features (panel b). Indeed, textural features present intermediate color hues between light yellow and dark blue, indicating whether the specific textural feature was removed according to the preprocessing parameter. On the other hand, shape and first-order features mainly present color hues equal to light yellow, i.e., the feature was permanently eliminated regardless of the preprocessing performed, or dark blue, i.e., the feature always remained regardless of preprocessing. This result is in line with a previous study on myocardial radiomic features derived from T1 and T2 mapping [37], showing that textural features estimate has a greater sensitivity to resampling voxel size and discretization bin width than shape or first-order features estimate.

For both effects A (i.e., varying resampling voxel sizes, with fixed discretization bin width) and B (i.e., varying discretization bin widths, with fixed resampling voxel size), our results suggest a greater influence on correlation-based dimensionality reduction in resampling voxel size than discretization bin width (Table 3 and Table 5). In fact, the stability indices are, as a whole, greater for effect B than for effect A, indicating how discretization bin width has less influence on Pearson’s as well as Spearman’s correlation analysis and the subsequent dimensionality reduction. As expected, we confirmed that features belonging to the shape and first-order classes are not sensitive to the change in discretization bin width, given that these radiomic features (except Entropy and Uniformity) were estimated before discretization under the IBSI recommendations [33] (panels (a) and (b) of Figure 4, Figure 5, Figure 8, and Figure 9).

Digital image filters can be applied before radiomic features extraction to detect and emphasize tissue characteristics different from those usually obtained from original images. In this regard, the IBSI has proposed a new reference manual to define and standardize the implementation of image filters in radiomics software [34]. Given that filtering can actually modify (even in a substantial way) T1 and T2 maps, we observed a relevant sensitivity of both PCC- and SCC-based dimensionality reduction to filtering. Although the percentage of remaining features is similar when using different filters, the stability indices were less than 0.5, indicating that the subsets of selected features were composed of different features.

A remarkable difference between T1 and T2 maps in sensitivity of collinearity analysis and dimensionality reduction to preprocessing was found. Performing the correlation-based dimensionality reduction on radiomic features from T2 maps was characterized by lower sensitivity to voxel size resampling and discretization than radiomic features from T1 maps. Regardless of whether PCC or SCC is used, the percentage of T2-derived features eliminated by the dimensionality reduction procedure is less than the percentage of removed T1-derived radiomic features. In addition, the remaining subsets of T2-derived features showed greater stability than the corresponding subsets of T1-derived features. These results, along with the previous findings by Marfisi et al. [37], also support the use of T2 mapping as a potential useful tool to describe myocardial structural anomalies in patients with HCM. So far, only T1 mapping has been used in previous HCM radiomic research, primarily due to its capacity to detect myocardial fibrosis [47,48,49,72]. T2 maps, however, are regarded as the gold standard for assessing myocardial edema, a well-known adverse prognostic feature in HCM [73,74]. While T2 mapping cannot evaluate myocardial fibrosis per se, texture analyses have the potential to circumvent this constraint by revealing myocardial structural heterogeneity caused by myofibrillar disarray and fibrosis [46,75].

We acknowledge the following issues as potential study limitations. First, we focused only on T1 and T2 maps, albeit they are particularly suitable for radiomic analysis, given their quantitative nature. Additional studies will be necessary to investigate the effect of preprocessing on correlation-based dimensionality reduction in radiomic features for classical cine, LGE, and STIR sequences. In HCM patients, T1 maps are usually obtained before and after contrast administration to calculate extracellular volume, a surrogate marker of interstitial remodeling and interstitial fibrosis [47,48,72]. However, in the present study, we focused only on native T1 values, mainly considering the possibility of obtaining equivalent information from radiomics analysis of non-contrast images to gadolinium-enhanced images. Indeed, avoiding contrast administration is a hugely desirable prospect currently under investigation.

Second, even though we only looked at single-slice T1 and T2 mapping acquisitions, myocardial alterations in HCM patients may also affect visibly non-hypertrophied cardiac parts. Consequently, a whole-heart coverage might offer a more thorough assessment of disease load and improve the diagnostic effectiveness of cardiac MRI. However, evaluating a single ROI on the mid-cavity short-axis map for a global/diffuse illness is considered sufficient [76]. Our technical study’s primary objective was to assess how voxel size resampling and discretization affected radiomic characteristics calculated from standard cardiac T1 and T2 mapping. As a result, we concentrated on a single short-axis slice at a location where myocardial changes were thought to be more severe, and the thickness of the myocardium was at its maximum. This allowed us to obtain minimum partial volume effects, which can significantly affect regions of myocardial segments that are thinner. Furthermore, given that this is a retrospective study with participants who had been referred for clinical or routine cardiac MRI, it is crucial to avoid having excessively lengthy acquisition times, especially for patients who are not cooperative.

Third, we included only twenty-six patients with HCM, representing a homogeneous group with the same pathology. Nonetheless, the findings of this technical investigation may be helpful and prodromic for future clinical studies, which should enroll a larger number of participants and include control subjects to specifically assess the clinical potential of radiomic analysis of T1 and T2 maps in HCM patients. This could lead to a better understanding of the role of CMR in differentiating HCM from hypertensive heart disease and other cardiomyopathies and in discriminating different genotypes of HCM, as well as in assessing arrhythmic risk in these patients [44,47,48].

Finally, our results depend on the cut-off thresholds defined for PCC and SCC. We decided to use the most chosen thresholds from previous studies that have performed correlation-based dimensionality reduction in a machine learning workflow (i.e., 0.8 for |PCC| and 0.9 for |SCC|) to understand this procedure’s sensitivity to preprocessing in a real scenario. Studying the changes in dimensionality reduction as the cut-off threshold changes was beyond the scope of this work.

## 5. Conclusions

In this HCM study, using radiomic features extracted from T1 and T2 maps, we observed a moderate sensitivity of collinearity analysis and correlation-based dimensionality reduction to some conventional image preprocessing procedures. While, as a whole, this effect is relatively moderate for voxel size resampling and discretization, it is remarkable when considering filtering. Moreover, correlation-based dimensionality reduction is less sensitive to preprocessing when considering radiomic features from T2 compared with T1 maps. Our findings further confirm the effect of preprocessing in radiomic analyses, with the consequent need of considering it toward a standardization of methods and when comparing data/results from different clinical studies.

## Figures and Tables

**Table 1:** Clinical and cardiac MRI-derived characteristics (mean (standard deviation)) of twenty-six patients with HCM.

Age (years)	66 (11)	
Myocardial thickness (mm)	19 (3)	
LGE	19/26	
	LV	RV
ED volume (mL/m^2^)	74 (15)	61 (13)
ES volume (mL/m^2^)	23 (14)	23 (6)
Stroke volume (mL/m^2^)	51 (13)	38 (9)
Ejection fraction (%)	70 (15)	63 (6)

ED: end diastolic, ES: end systolic, LGE: late gadolinium enhancement, LV: left ventricle, RV: right ventricle.

**Table 2:** Collinearity analysis and correlation-based dimensionality reduction for T1 maps. In the column “# of CC”, the number of pair-wise correlation coefficients that were significant and, in absolute value, greater than the predefined threshold is reported (see Section 2.5 for details). In the column “% of remaining features”, the percentage of remaining features after the correlation-based dimensionality reduction is reported.

Effect A—Varying Resampling Voxel Size Values in [1.8, 1.9, 2.0, 2.1, 2.2, 2.3, 2.4] mm, with Fixed Discretization BW				
	Pearson-correlation-based dimensionality reduction		Spearman-correlation-based dimensionality reduction	
Discretization BW (ms)	# of CC	% of remaining features	# of CC	% of remaining features
3.60	[903 774 660 793 806 778 659]	[21 24 24 22 23 23 23]	[497 447 507 438 491 461 392]	[28 28 29 27 27 33 31]
3.95	[882 799 676 800 839 851 655]	[21 24 23 23 22 22 24]	[490 489 510 416 466 498 413]	[29 29 26 29 29 31 29]
4.30	[880 824 680 778 866 825 743]	[23 24 22 23 22 24 22]	[476 499 554 391 490 461 442]	[30 28 29 29 27 31 30]
4.65	[878 816 682 860 810 831 699]	[22 24 24 20 23 24 23]	[498 477 502 479 443 462 442]	[31 30 30 29 32 33 31]
5.00	[900 806 707 840 877 824 712]	[23 24 21 22 23 23 23]	[491 444 538 484 499 486 446]	[31 29 27 31 29 30 29]
5.35	[894 829 712 790 881 878 736]	[23 24 24 22 23 23 23]	[491 492 495 419 426 455 423]	[31 32 29 31 29 30 27]
5.70	[892 819 739 821 862 824 729]	[26 24 24 24 23 24 23]	[501 475 539 447 442 440 434]	[29 30 31 32 30 31 30]
6.05	[908 846 702 777 888 841 731]	[24 23 26 23 22 24 20]	[503 483 518 430 464 459 446]	[30 31 31 32 32 33 28]
6.40	[899 864 714 839 857 843 698]	[24 24 27 22 23 24 23]	[489 463 523 446 474 464 438]	[31 31 31 32 30 32 30]
**Effect B—Varying Discretization BW Values in [3.60, 3.95, 4.30, 4.65, 5.00, 5.35, 5.70, 6.05, 6.40] ms, with Fixed Resampling Voxel Size**				
	Pearson-correlation-based dimensionality reduction		Spearman-correlation-based dimensionality reduction	
Resampling voxel size (mm)	# of CC	% of remaining features	# of CC	% of remaining features
1.8	[903 882 880 878 900 894 892 908 899]	[21 21 23 22 23 23 26 24 24]	[497 490 476 498 491 491 501 503 489]	[28 29 30 31 31 31 29 30 31]
1.9	[774 799 824 816 806 829 819 846 864]	[24 24 24 24 24 24 24 23 24]	[447 489 499 477 444 492 475 483 463]	[28 29 28 30 29 32 30 31 31]
2.0	[660 676 680 682 707 712 739 702 714]	[24 23 22 24 21 24 24 26 27]	[507 510 554 502 538 495 539 518 523]	[29 26 29 30 27 29 31 31 31]
2.1	[793 800 778 860 840 790 821 777 839]	[22 23 23 20 22 22 24 23 22]	[438 416 391 479 484 419 447 430 446]	[27 29 29 29 31 31 32 32 32]
2.2	[806 839 866 810 877 881 862 888 857]	[23 22 22 23 23 23 23 22 23]	[491 466 490 443 499 426 442 464 474]	[27 29 27 32 29 29 30 32 30]
2.3	[778 851 825 831 824 878 824 841 843]	[23 22 24 24 23 23 24 24 24]	[461 498 461 462 486 455 440 459 464]	[33 31 31 33 30 30 31 33 32]
2.4	[659 655 743 699 712 736 729 731 698]	[23 24 22 23 23 23 23 20 23]	[392 413 442 442 446 423 434 446 438]	[31 29 30 31 29 27 30 28 30]
**Effect C—Varying Filtering, with Fixed Resampling Voxel Size (2.1 mm) and Discretization BW (6 ms)**				
	Pearson-correlation-based dimensionality reduction		Spearman-correlation-based dimensionality reduction	
Filter	# of CC	% of remaining features	# of CC	% of remaining features
Original	744	20	426	29
Gradient	1244	20	1116	28
Square	958	21	462	28
SquareRoot	816	20	590	27
Wavelet-HH	891	18	726	26
Wavelet-HL	835	21	432	30
Wavelet-LH	826	22	469	28
Wavelet-LL	584	24	483	29

BW: bin width, CC: correlation coefficient, HH: horizontal and vertical high-pass filters, HL: horizontal high-pass filter and vertical low-pass filter, LH: horizontal low-pass filter and vertical high-pass filter, LL: horizontal and vertical low-pass filters.

**Table 3:** Stability indices for T1 maps.

Effect A—Varying Resampling Voxel Size Values in [1.8, 1.9, 2.0, 2.1, 2.2, 2.3, 2.4] mm), with Fixed Discretization BW		
Discretization BW (ms)	Pearson-correlation-based dimensionality reduction stability	Spearman-correlation-based dimensionality reduction stability
360	0.66	0.60
395	0.66	0.60
430	0.63	0.63
465	0.64	0.58
500	0.60	0.61
535	0.65	0.60
570	0.65	0.60
605	0.66	0.58
640	0.64	0.57
**Effect B—Varying Discretization BW Values in [3.60, 3.95, 4.30, 4.65, 5.00, 5.35, 5.70, 6.05, 6.40] ms, with Fixed Resampling Voxel Size**		
Resampling voxel size (mm)	Pearson’s correlation-based dimensionality reduction stability	Spearman’s correlation-based dimensionality reduction stability
1.8	0.69	0.66
1.9	0.85	0.68
2.0	0.77	0.67
2.1	0.65	0.71
2.2	0.69	0.71
2.3	0.77	0.66
2.4	0.70	0.74
**Effect C—Varying Filtering, with Fixed Resampling Voxel Size (2.1 mm) and Discretization BW (6 ms)**		
	Pearson-correlation-based dimensionality reduction stability	Spearman-correlation-based dimensionality reduction stability
Filtering	0.38	0.42

BW: bin width.

**Table 4:** Collinearity analysis and correlation-based dimensionality reduction for T2 maps. In the column “# of CC”, the number of pair-wise correlation coefficients that were significant and, in absolute value, greater than the predefined threshold is reported (see Section 2.5 for details). In the column “% of remaining features”, the percentage of remaining features after the correlation-based dimensionality reduction is reported.

Effect A—Varying Resampling Voxel Size Values in [1.8, 1.9, 2.0, 2.1, 2.2, 2.3, 2.4] mm, with Fixed Discretization BW				
	Pearson-correlation-based dimensionality reduction		Spearman-correlation-based dimensionality reduction	
Discretization BW (ms)	# of CC	% of remaining features	# of CC	% of remaining features
0.49	[553 579 546 567 534 546 594]	[24 23 21 22 23 22 22]	[417 445 422 453 443 399 410]	[32 32 34 31 30 34 34]
0.50	[570 582 564 570 576 538 605]	[24 21 21 22 23 23 21]	[414 432 438 467 431 393 479]	[33 32 34 32 34 31 32]
0.51	[574 594 547 583 572 544 607]	[24 22 22 20 23 22 19]	[403 460 432 467 444 380 520]	[34 33 33 31 33 32 32]
0.52	[564 574 540 583 557 550 609]	[23 22 22 22 26 21 21]	[386 431 418 455 406 412 467]	[34 32 33 32 35 30 32]
0.53	[558 582 562 555 564 567 599]	[24 26 21 21 24 20 22]	[419 439 446 435 418 398 487]	[31 32 33 32 35 32 32]
0.54	[572 589 570 568 572 554 621]	[23 22 21 21 22 21 19]	[399 442 453 442 430 419 444]	[33 35 31 33 34 31 33]
0.55	[582 579 564 575 563 555 572]	[23 22 22 21 24 21 23]	[415 426 414 429 435 417 422]	[32 30 32 32 35 31 33]
0.56	[555 599 559 580 572 558 605]	[24 22 22 22 24 21 20]	[413 467 422 448 426 408 450]	[31 31 35 35 34 32 32]
0.57	[583 591 562 574 576 526 597]	[24 21 23 21 26 21 21]	[413 426 442 456 435 405 472 ]	[32 34 31 31 33 34 31]
**Effect B—Varying Discretization BW Values in [0.49, 0.50, 0.51, 0.52, 0.53, 0.54, 0.55, 0.56, 0.57] ms, with Fixed Resampling Voxel Size**				
	Pearson-correlation-based dimensionality reduction		Spearman-correlation-based dimensionality reduction	
Resampling voxel size (mm)	# of CC	% of remaining features	# of CC	% of remaining features
1.8	[553 570 574 564 558 572 582 555 583]	[24 24 24 23 24 23 23 24 24]	[417 414 403 386 419 399 415 413 413]	[32 33 34 34 31 33 32 31 32]
1.9	[579 582 594 574 582 589 579 599 591]	[23 21 22 22 26 22 22 22 21]	[445 432 460 431 439 442 426 467 426]	[32 32 33 32 32 35 30 31 34]
2.0	[546 564 547 540 562 570 564 559 562]	[21 21 22 22 21 21 22 22 23]	[422 438 432 418 446 453 414 422 442]	[34 34 33 33 33 31 32 35 31]
2.1	[567 570 583 583 555 568 575 580 574]	[22 22 20 22 21 21 21 22 21]	[453 467 467 455 435 442 429 448 456]	[31 32 31 32 32 33 32 35 31]
2.2	[534 576 572 557 564 572 563 572 576]	[23 23 23 26 24 22 24 24 26]	[443 431 444 406 418 430 435 426 435]	[30 34 33 35 35 34 35 34 33]
2.3	[546 538 544 550 567 554 555 558 526]	[22 23 22 21 20 21 21 21 21]	[399 393 380 412 398 419 417 408 405]	[34 31 32 30 32 31 31 32 34]
2.4	[594 605 607 609 599 621 572 605 597]	[22 21 19 21 22 19 23 20 21]	[410 479 520 467 487 444 422 450 472]	[34 32 32 32 32 33 33 32 31]
**Effect C—Varying Filtering, with Fixed Resampling Voxel Size (2.1 mm) and Discretization BW (0.56 ms)**				
	Pearson-correlation-based dimensionality reduction		Spearman-correlation-based dimensionality reduction	
Filter	# of CC	% of remaining features	# of CC	% of remaining features
Original	551	21	429	31
Gradient	1487	19	1138	28
Square	817	20	564	36
SquareRoot	552	20	447	25
Wavelet-HH	841	17	738	24
Wavelet-HL	753	19	688	24
Wavelet-LH	870	20	469	22
Wavelet-LL	563	18	412	29

**Table 5:** Stability indices for T2 maps.

Effect A—Varying Resampling Voxel Size Values in [1.8, 1.9, 2.0, 2.1, 2.2, 2.3, 2.4] mm, with Fixed Discretization BW		
Discretization BW (ms)	Pearson-correlation-based dimensionality reduction stability	Spearman-correlation-based dimensionality reduction stability
0.49	0.75	0.73
0.50	0.69	0.70
0.51	0.70	0.68
0.52	0.74	0.70
0.53	0.69	0.68
0.54	0.67	0.70
0.55	0.72	0.71
0.56	0.74	0.71
0.57	0.69	0.65
**Effect B—Varying Discretization BW Values in [0.49, 0.50, 0.51, 0.52, 0.53, 0.54, 0.55, 0.56, 0.57] ms, with Fixed Resampling Voxel Size**		
Resampling voxel size (mm)	Pearson-correlation-based dimensionality reduction stability	Spearman-correlation-based dimensionality reduction stability
1.8	0.81	0.86
1.9	0.89	0.79
2.0	0.88	0.81
2.1	0.81	0.77
2.2	0.86	0.79
2.3	0.84	0.79
2.4	0.84	0.84
**Effect C—Varying Filtering, with Fixed Resampling Voxel Size (2.1 mm) and Discretization BW (0.56 ms)**		
	Pearson-correlation-based dimensionality reduction stability	Spearman-correlation-based dimensionality reduction stability
Filtering	0.40	0.43

BW: bin width.

## Data Availability

The datasets generated during and/or analyzed during the current study are available from the corresponding author on reasonable request.

## Supplementary Materials

The following are available online at https://www.mdpi.com/article/10.3390/bioengineering10010080/s1.
