# Supplementary material for: Collinearity and Dimensionality Reduction in Radiomics: Effect of Preprocessing Parameters in Hypertrophic Cardiomyopathy Magnetic Resonance T1 and T2 Mapping
Source: Bioengineering (Basel). 2023 Jan 6;10(1):80. doi: 10.3390/bioengineering10010080 (PMC9854492; doi:10.3390/bioengineering10010080)
Supplement: Supplementary file 1 [file bioengineering-10-00080-s001.zip › bioengineering-2110933-supplementary.pdf]

Supplementary Material

Collinearity and dimensionality reduction in radiomics: effect of preprocessing parameters in hypertrophic cardiomyopathy magnetic resonance T1 and T2 mapping

Chiara Marzi[1], Daniela Marfisi[2], Andrea Barucci[1], Jacopo Del Meglio[3], Alessio Lilli[3], Claudio Vignali[4], Mario Mascalchi[5], Giancarlo Casolo[3] , Stefano Diciotti[6], Antonio Claudio Traino[2], Carlo Tessa[7,‡] and Marco Giannelli[2,‡,*]

[1] Institute of Applied Physics "Nello Carrara" (IFAC), Council of National Research (CNR), Sesto Fiorentino, Florence, Italy
[2] Unit of Medical Physics, Pisa University Hospital "Azienda Ospedaliero-Universitaria Pisana", Via Roma 67, 56126, Pisa, Italy
[3] Unit of Cardiology, Azienda USL Toscana Nord Ovest, Versilia Hospital, 55041 Lido di Camaiore, Italy.
[4] Unit of Radiology, Azienda USL Toscana Nord Ovest, Versilia Hospital, 55041 Lido di Camaiore, Italy.
[5] Department of Experimental and Clinical Biomedical Sciences "Mario Serio", University of Florence, 50121, Florence, Italy.
[6] Department of Electrical, Electronic, and Information Engineering "Guglielmo Marconi", University of Bologna, 47522, Cesena, Italy.
[7] Unit of Radiology, Azienda USL Toscana Nord Ovest, Apuane Hospital, 54100, Massa, Italy
* Correspondence: m.giannelli@ao-pisa.toscana.it.
‡ These authors contributed equally to this work.

Table S1. Matching between numerical label, used in supplementary figures, and radiomic feature name, in accordance with PyRadiomics (https://pyradiomics.readthedocs.io/en/latest/features.html). No shape features were included in our analysis in case of filtering.

# for original maps	# for filtered maps	Radiomic features
0	NA	shape2D_Elongation
1	NA	shape2D_MajorAxisLength
2	NA	shape2D_MaximumDiameter
3	NA	shape2D_MeshSurface
4	NA	shape2D_MinorAxisLength
5	NA	shape2D_Perimeter
6	NA	shape2D_PerimeterSurfaceRatio
7	NA	shape2D_PixelSurface
8	NA	shape2D_Sphericity
9	0	firstorder_Mean
10	1	firstorder_Variance
11	2	firstorder_Skewness
12	3	firstorder_Kurtosis
13	4	firstorder_Median
14	5	firstorder_Minimum
15	6	firstorder_10Percentile
16	7	firstorder_90Percentile

17	8	firstorder_Maximum
18	9	firstorder_InterquartileRange
19	10	firstorder_MeanAbsoluteDeviation
20	11	firstorder_RobustMeanAbsoluteDeviation
21	12	firstorder_Energy
22	13	firstorder_RootMeanSquared
23	14	firstorder_Entropy
24	15	firstorder_Uniformity
25	16	glcm_MaximumProbability
26	17	glcm_JointAverage
27	18	glcm_SumSquares
28	19	glcm_JointEntropy
29	20	glcm_DifferenceAverage
30	21	glcm_DifferenceVariance
31	22	glcm_DifferenceEntropy
32	23	glcm_SumEntropy
33	24	glcm_JointEnergy
34	25	glcm_Contrast
35	26	glcm_Id
36	27	glcm_Idn
37	28	glcm_Idm
38	29	glcm_Idmn
39	30	glcm_InverseVariance
40	31	glcm_Correlation
41	32	glcm_Autocorrelation
42	33	glcm_ClusterTendency
43	34	glcm_ClusterShade
44	35	glcm_ClusterProminence
45	36	glcm_Imc1
46	37	glcm_Imc2
47	38	glrlm_GrayLevelNonUniformity
48	39	glrlm_GrayLevelNonUniformityNormalized
49	40	glrlm_GrayLevelVariance
50	41	glrlm_HighGrayLevelRunEmphasis
51	42	glrlm_LongRunEmphasis
52	43	glrlm_LongRunHighGrayLevelEmphasis
53	44	glrlm_LongRunLowGrayLevelEmphasis
54	45	glrlm_LowGrayLevelRunEmphasis
55	46	glrlm_RunEntropy
56	47	glrlm_RunLengthNonUniformity
57	48	glrlm_RunLengthNonUniformityNormalized
58	49	glrlm_RunPercentage

59	50	glrlm_RunVariance
60	51	glrlm_ShortRunEmphasis
61	52	glrlm_ShortRunHighGrayLevelEmphasis
62	53	glrlm_ShortRunLowGrayLevelEmphasis
63	54	glszm_GrayLevelNonUniformity
64	55	glszm_GrayLevelNonUniformityNormalized
65	56	glszm_GrayLevelVariance
66	57	glszm_HighGrayLevelZoneEmphasis
67	58	glszm_LargeAreaEmphasis
68	59	glszm_LargeAreaHighGrayLevelEmphasis
69	60	glszm_LargeAreaLowGrayLevelEmphasis
70	61	glszm_LowGrayLevelZoneEmphasis
71	62	glszm_SizeZoneNonUniformity
72	63	glszm_SizeZoneNonUniformityNormalized
73	64	glszm_SmallAreaEmphasis
74	65	glszm_SmallAreaHighGrayLevelEmphasis
75	66	glszm_SmallAreaLowGrayLevelEmphasis
76	67	glszm_ZoneEntropy
77	68	glszm_ZonePercentage
78	69	glszm_ZoneVariance
79	70	ngtdm_Busyness
80	71	ngtdm_Coarseness
81	72	ngtdm_Complexity
82	73	ngtdm_Contrast
83	74	ngtdm_Strength
84	75	gldm_DependenceEntropy
85	76	gldm_DependenceNonUniformity
86	77	gldm_DependenceNonUniformityNormalized
87	78	gldm_DependenceVariance
88	79	gldm_GrayLevelNonUniformity
89	80	gldm_GrayLevelVariance
90	81	gldm_HighGrayLevelEmphasis
91	82	gldm_LargeDependenceEmphasis
92	83	gldm_LargeDependenceHighGrayLevelEmphasis
93	84	gldm_LargeDependenceLowGrayLevelEmphasis
94	85	gldm_LowGrayLevelEmphasis
95	86	gldm_SmallDependenceEmphasis
96	87	gldm_SmallDependenceHighGrayLevelEmphasis
97	88	gldm_SmallDependenceLowGrayLevelEmphasis

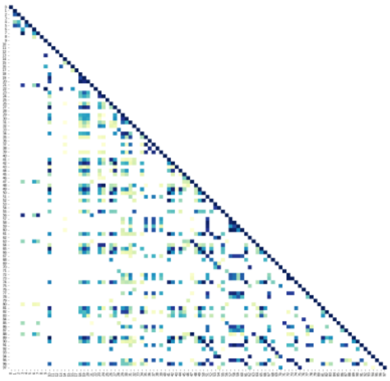

(a)

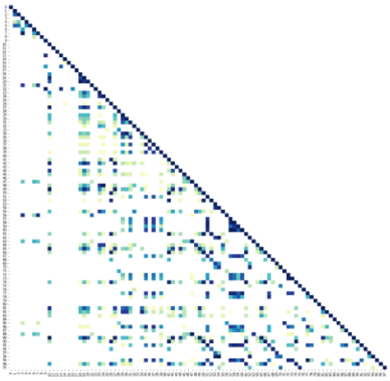

(b)

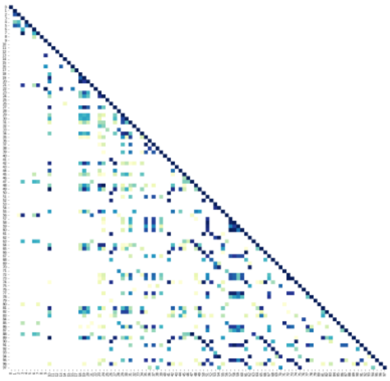

(c)

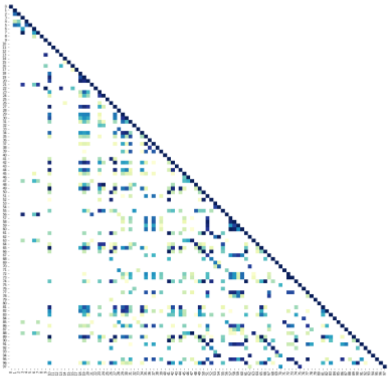

(d)

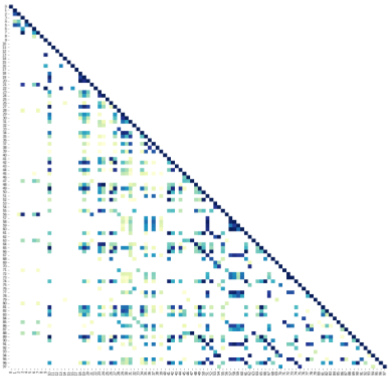

(e)

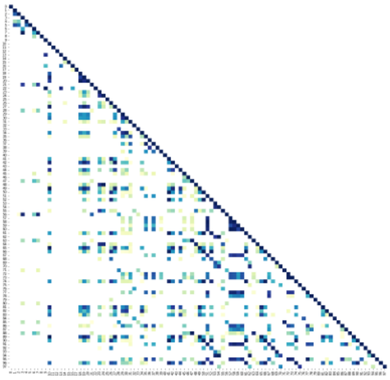

(f)

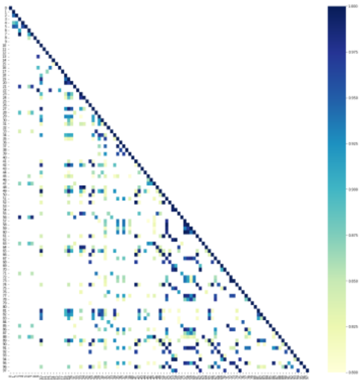

(g)

Figure S1. Examples of heatmaps of significant PCCs with absolute value greater than 0.8 for T1 maps, at fixed discretization bin width of 5.35 ms and resampling voxel size of 1.8 mm (S1a), 1.9 mm (S1b), 2.0 mm (S1c), 2.1 mm (S1d), 2.2 mm (S1e), 2.3 mm (S1f), and 2.4 mm (S1g). Numerical labels and related radiomic features are reported in Table S1.

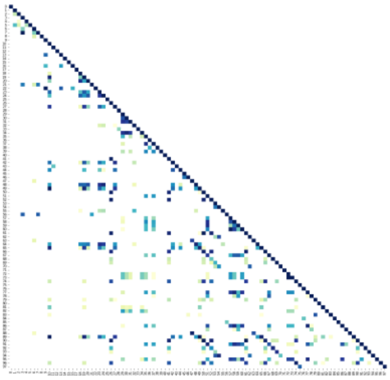

(a)

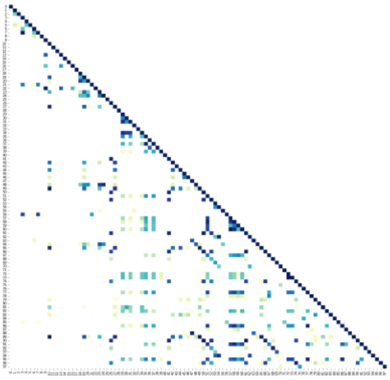

(b)

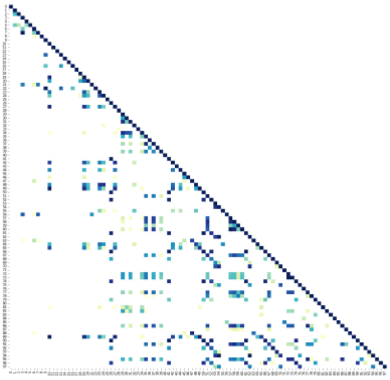

(c)

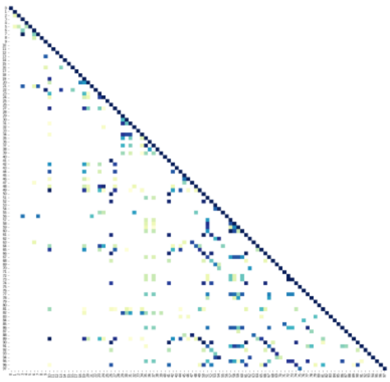

(d)

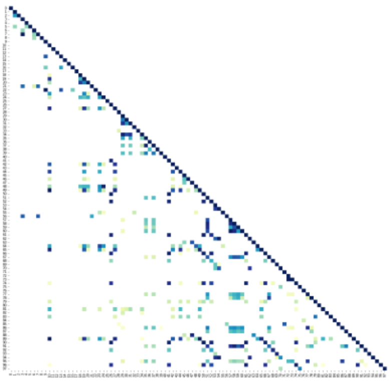

(e)

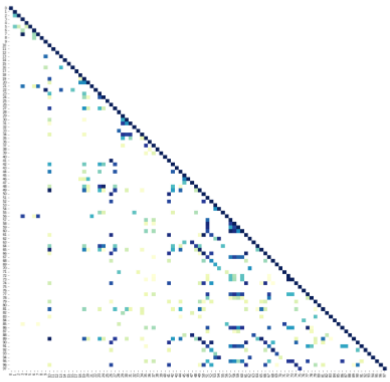

(f)

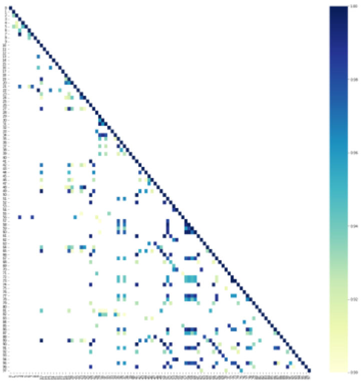

(g)

Figure S2. Examples of heatmaps of significant SCCs with absolute value greater than 0.9 for T1 maps, at fixed discretization bin width of 6.05 ms and resampling voxel size of 1.8 mm (S2a), 1.9 mm (S2b), 2.0 mm (S2c), 2.1 mm (S2d), 2.2 mm (S2e), 2.3 mm (S2f), and 2.4 mm (S2g). Numerical labels and related radiomic features are reported in Table S1.

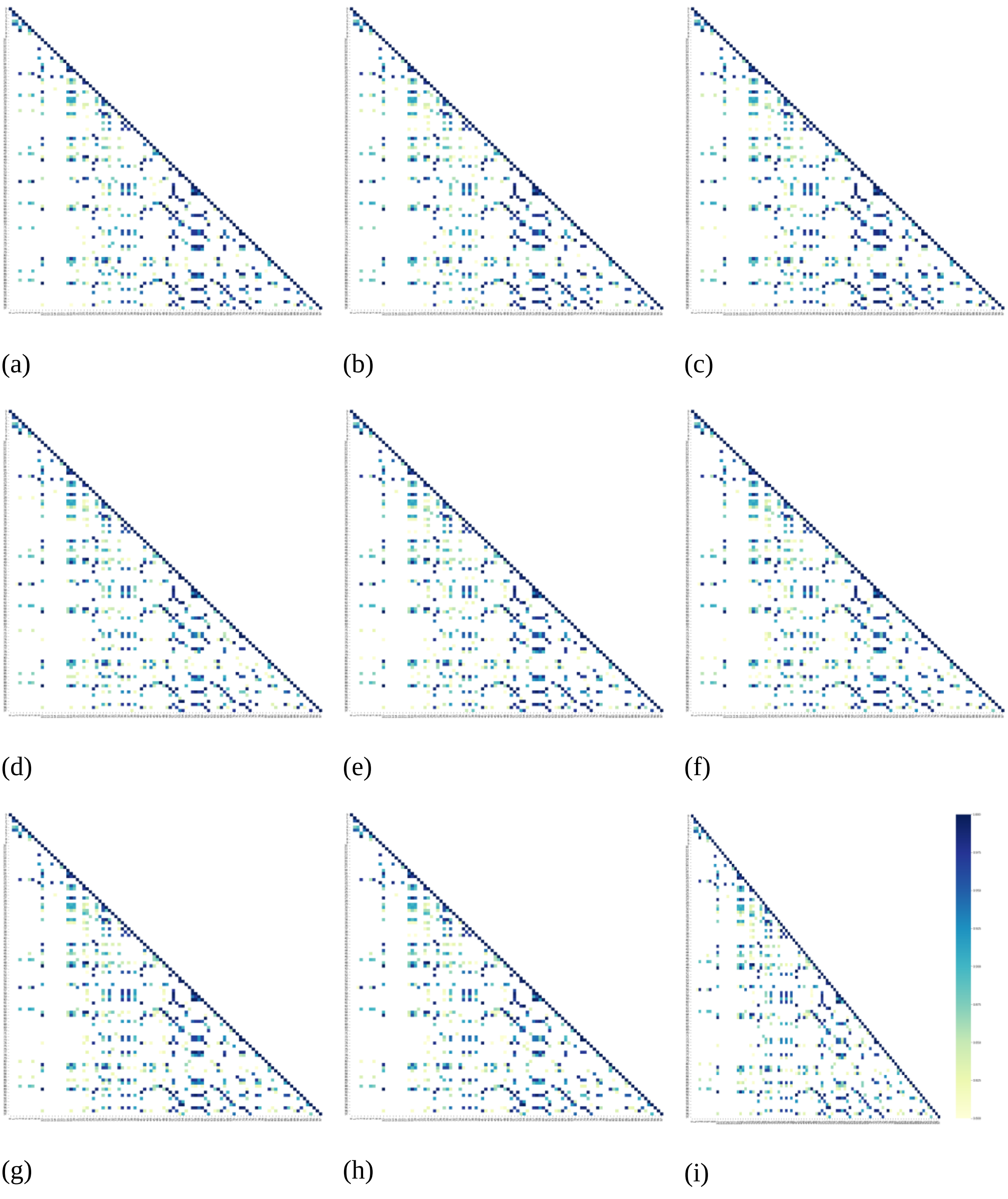

Figure S3. Examples of heatmaps of significant PCCs with absolute value greater than 0.8 for T1 maps, at fixed resampling voxel size of 2.0 mm and discretization bin width of 3.60 (S3a), 3.95 (S3b), 4.30 (S3c), 4.65 (S3d), 5.00 (S3e), 5.35 (S3f), 5.70 (S3g), 6.05 (S3h), and 6.40 (S3i). Numerical labels and related radiomic features are reported in Table S1.

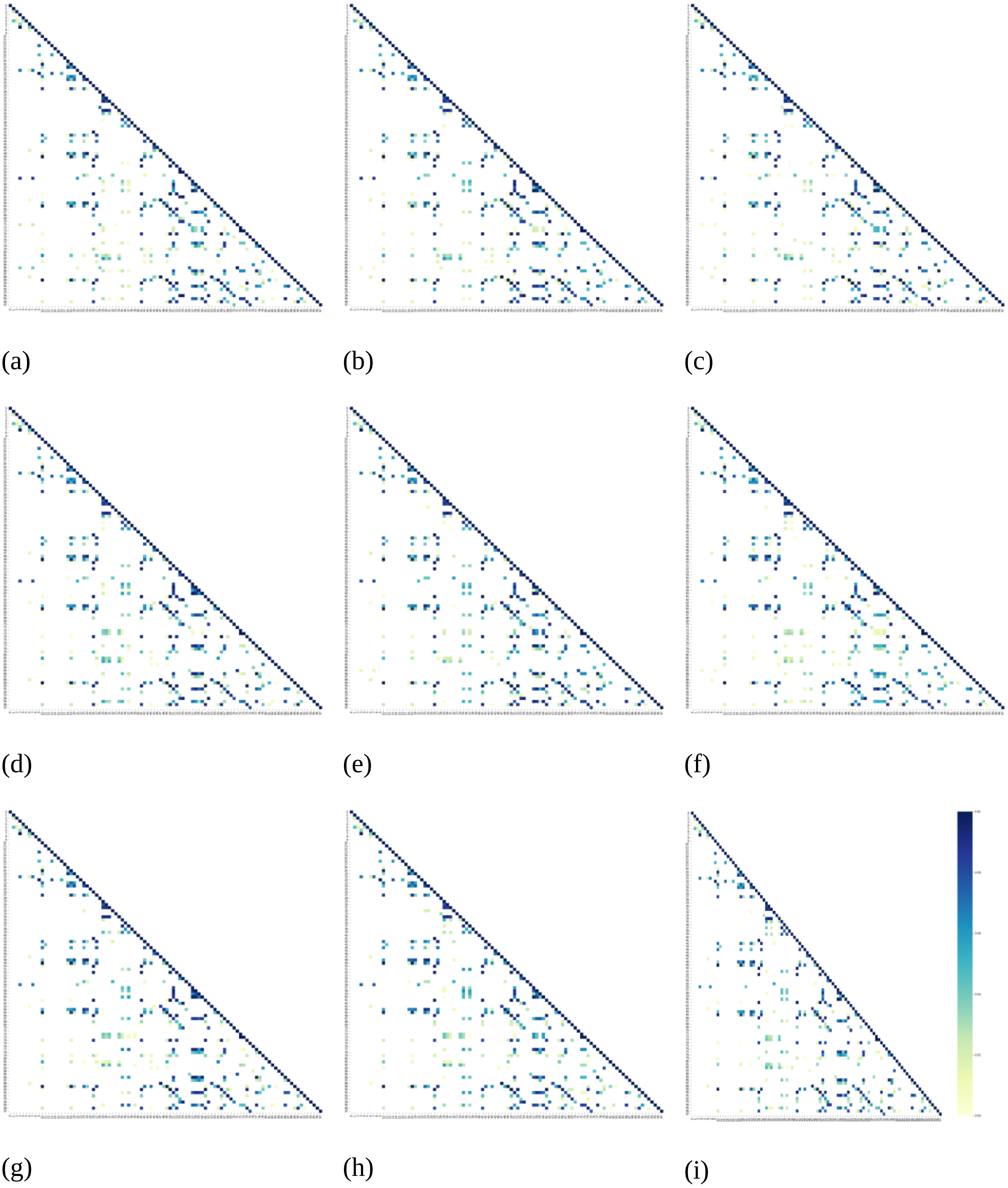

Figure S4. Examples of heatmaps of significant SCCs with absolute value greater than 0.9 for T1 maps, at fixed resampling voxel size of 1.8 mm and discretization bin width of 3.60 (S4a), 3.95 (S4b), 4.30 (S4c), 4.65 (S4d), 5.00 (S4e), 5.35 (S4f), 5.70 (S4g), 6.05 (S4h), and 6.40 (S4i). Numerical labels and related radiomic features are reported in Table S1.

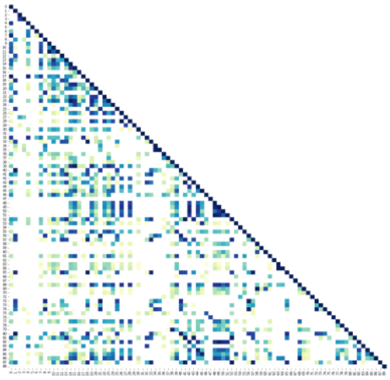

(a)

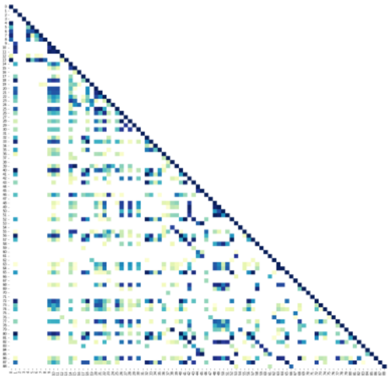

(b)

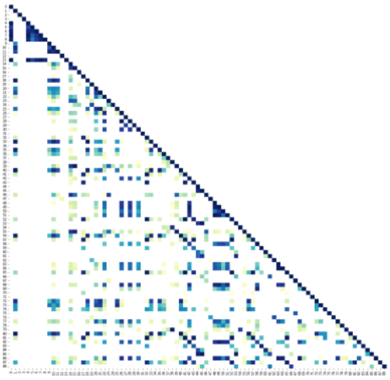

(c)

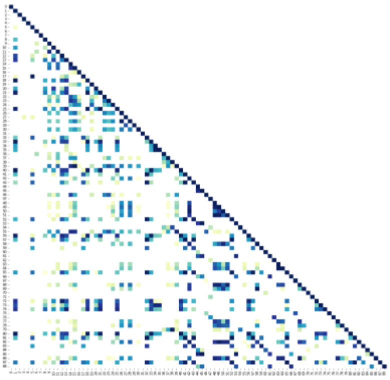

(d)

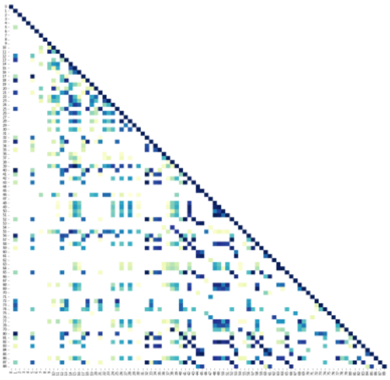

(e)

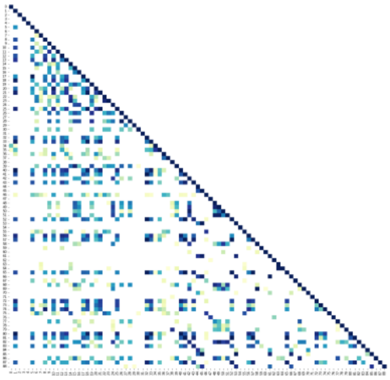

(f)

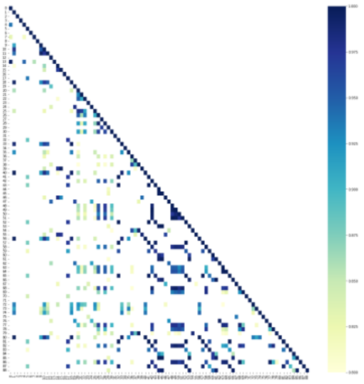

(g)

Figure S5. Heatmaps of significant PCCs with absolute value greater than 0.8 for T1 maps, at fixed resampling voxel size of 2.1 mm and discretization bin width of 6 ms, with varying filtering: gradient (S5a), square (S5b), square-root (S5c), wavelet-LH (S5d), wavelet-HL (S5e), wavelet-HH (S5f), and wavelet-LL (S5g). HH: horizontal and vertical high-pass filters, HL: horizontal high-pass filter and vertical low-pass filter, LH: horizontal low-pass filter and vertical high-pass filter, LL: horizontal and vertical low-pass filters. Numerical labels and related radiomic features are reported in Table S1.

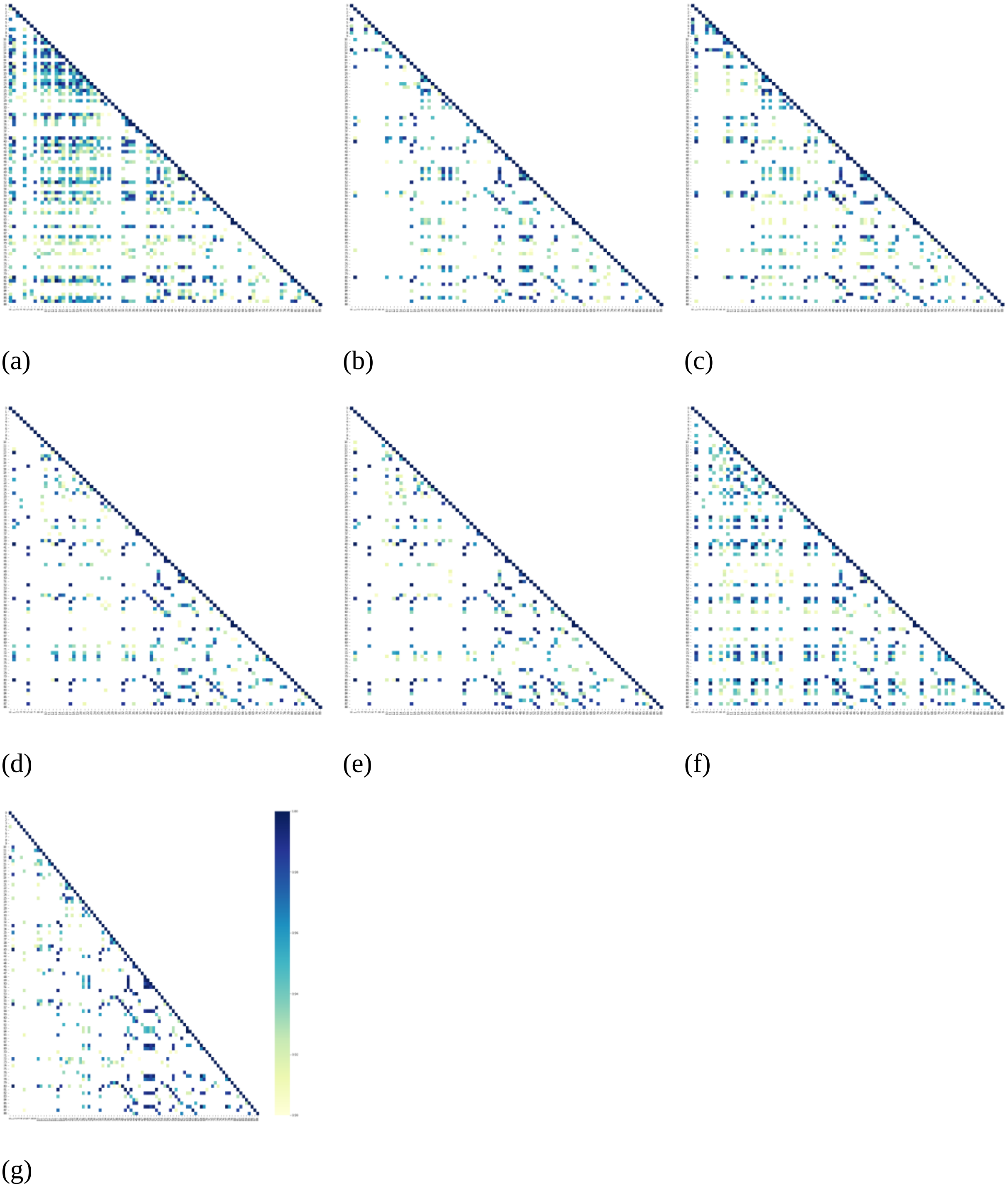

Figure S6. Heatmaps of significant SCCs with absolute value greater than 0.9 for T1 maps, at fixed resampling voxel size of 2.1 mm and discretization bin width of 6 ms, with varying filtering: gradient (S6a), square (S6b), square-root (S6c), wavelet-LH (S6d), wavelet-HL (S6e), wavelet-HH (S6f), and wavelet-LL (S6g). HH: horizontal and vertical high-pass filters, HL: horizontal high-pass filter and vertical low-pass filter, LH: horizontal low-pass filter and vertical high-pass filter, LL: horizontal and vertical low-pass filters. Numerical labels and related radiomic features are reported in Table S1.

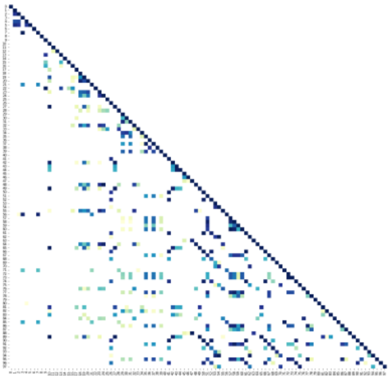

(a)

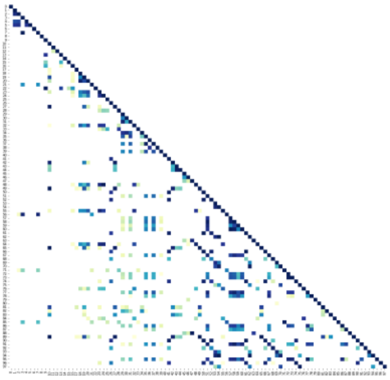

(b)

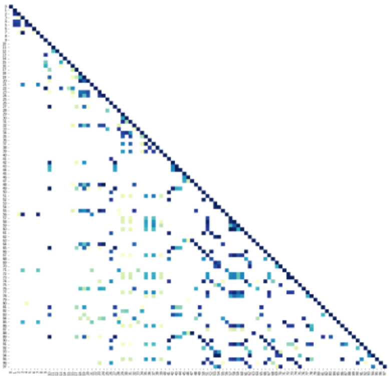

(c)

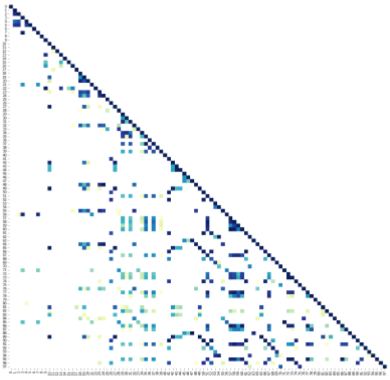

(d)

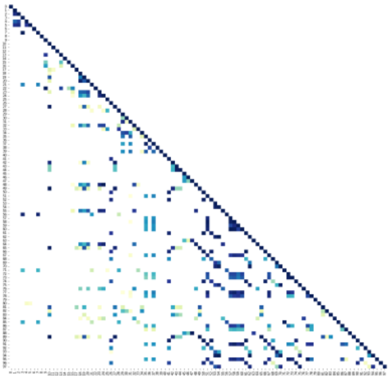

(e)

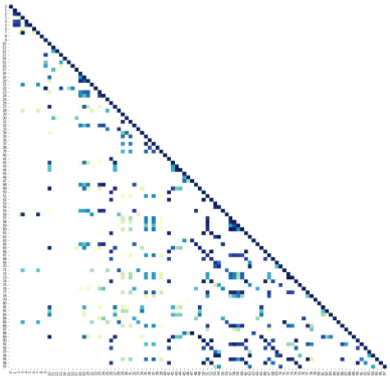

(f)

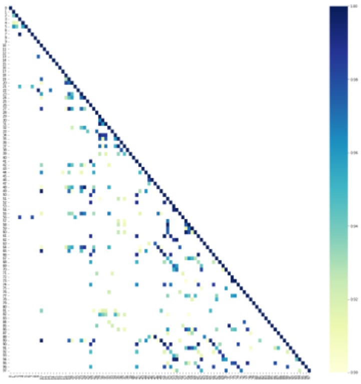

(g)

Figure S7. Examples of heatmaps of significant PCCs with absolute value greater than 0.8 for T2 maps, at fixed discretization bin width of 0.49 ms and resampling voxel size of 1.8 mm (S7a), 1.9 mm (S7b), 2.0 mm (S7c), 2.1 mm (S7d), 2.2 mm (S7e), 2.3 mm (S7f), and 2.4 mm (S7g). Numerical labels and related radiomic features are reported in Table S1.

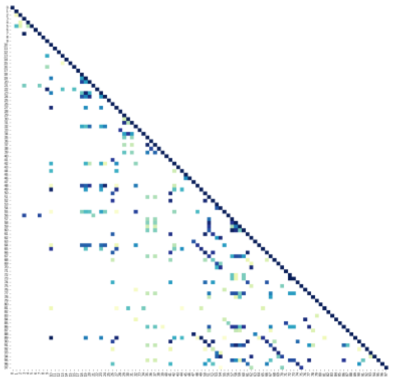

(a)

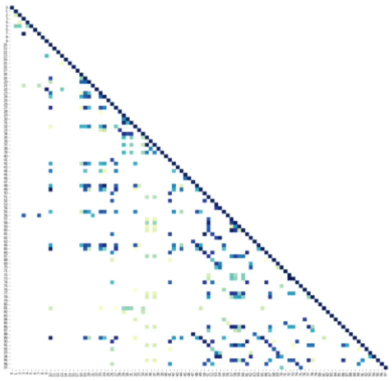

(b)

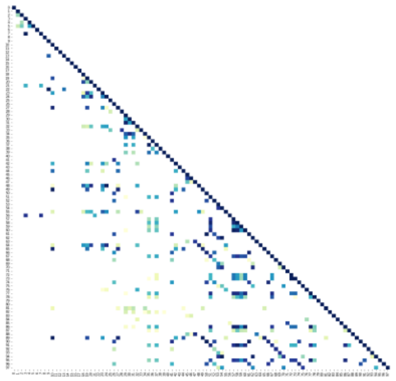

(c)

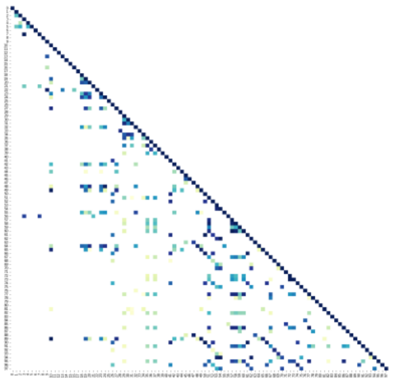

(d)

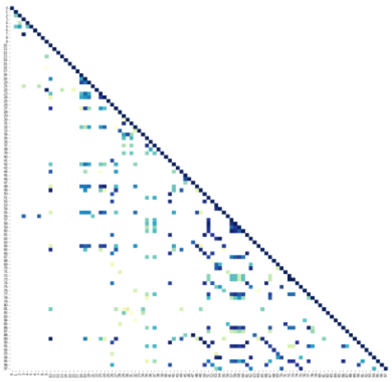

(e)

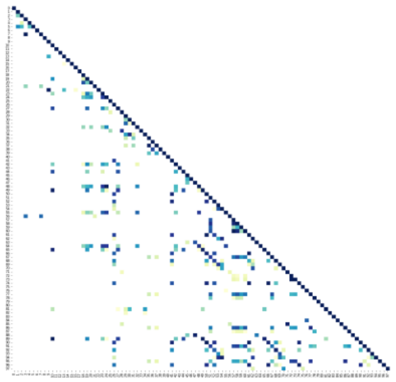

(f)

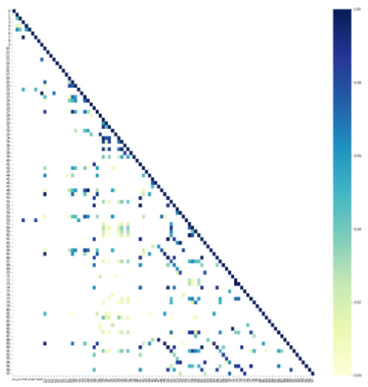

(g)

Figure S8. Examples of heatmaps of significant SCCs with absolute value greater than 0.9 for T2 maps, at fixed discretization bin width of 0.54 ms and resampling voxel size of 1.8 mm (S8a), 1.9 mm (S8b), 2.0 mm (S8c), 2.1 mm (S8d), 2.2 mm (S8e), 2.3 mm (S8f), and 2.4 mm (S8g). Numerical labels and related radiomic features are reported in Table S1.

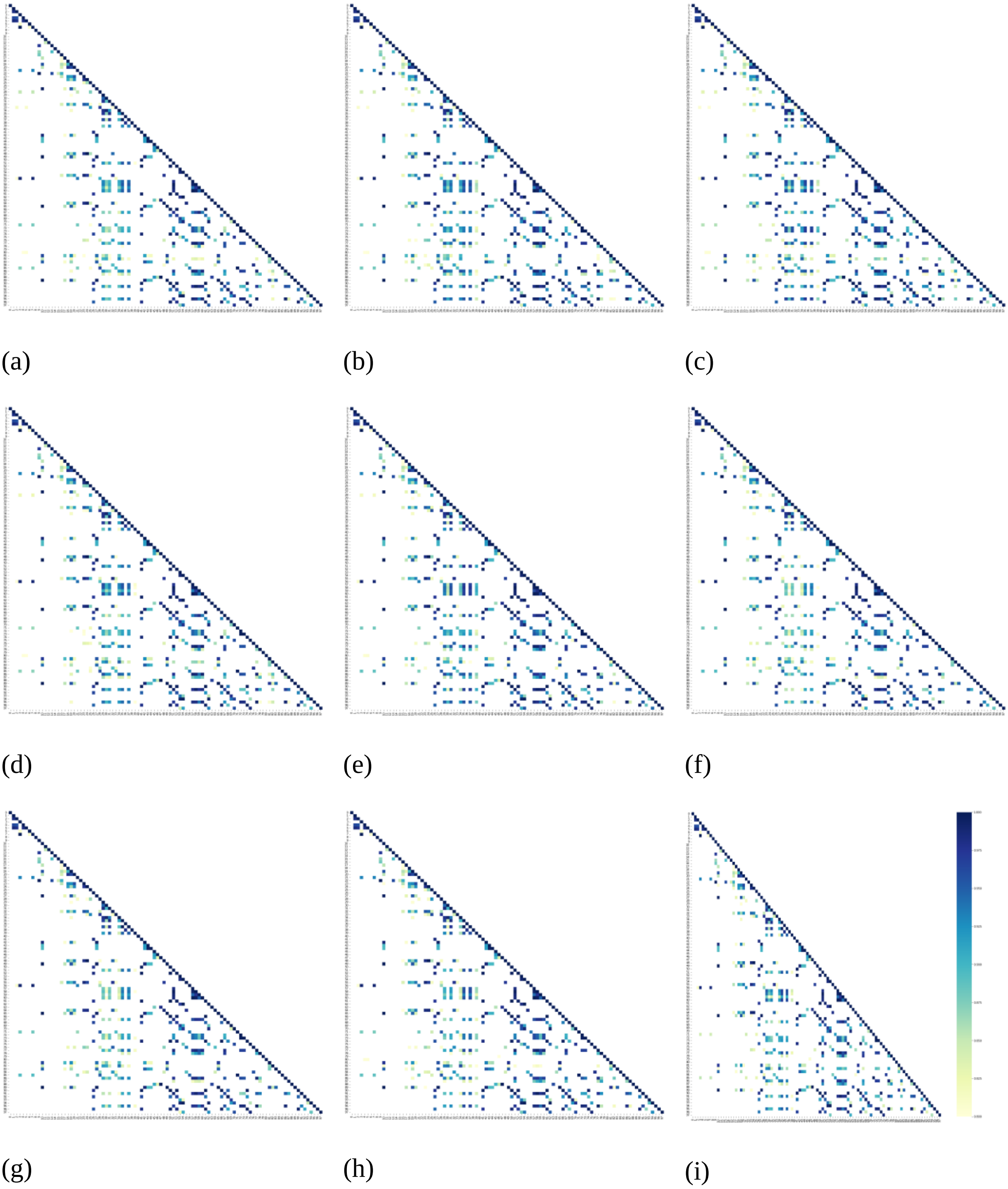

Figure S9. Examples of heatmaps of significant PCCs with absolute value greater than 0.8 for T2 maps, at fixed resampling voxel size of 2.4 mm and discretization bin width of 0.49 ms (S9a), 0.50 ms (S9b), 0.51 ms (S9c), 0.52 ms (S9d), 0.53 ms (S9e), 0.54 ms (S9f), 0.55 ms (S9g), 0.56 ms (S9h), and 0.57 ms (S9i). Numerical labels and related radiomic features are reported in Table S1.

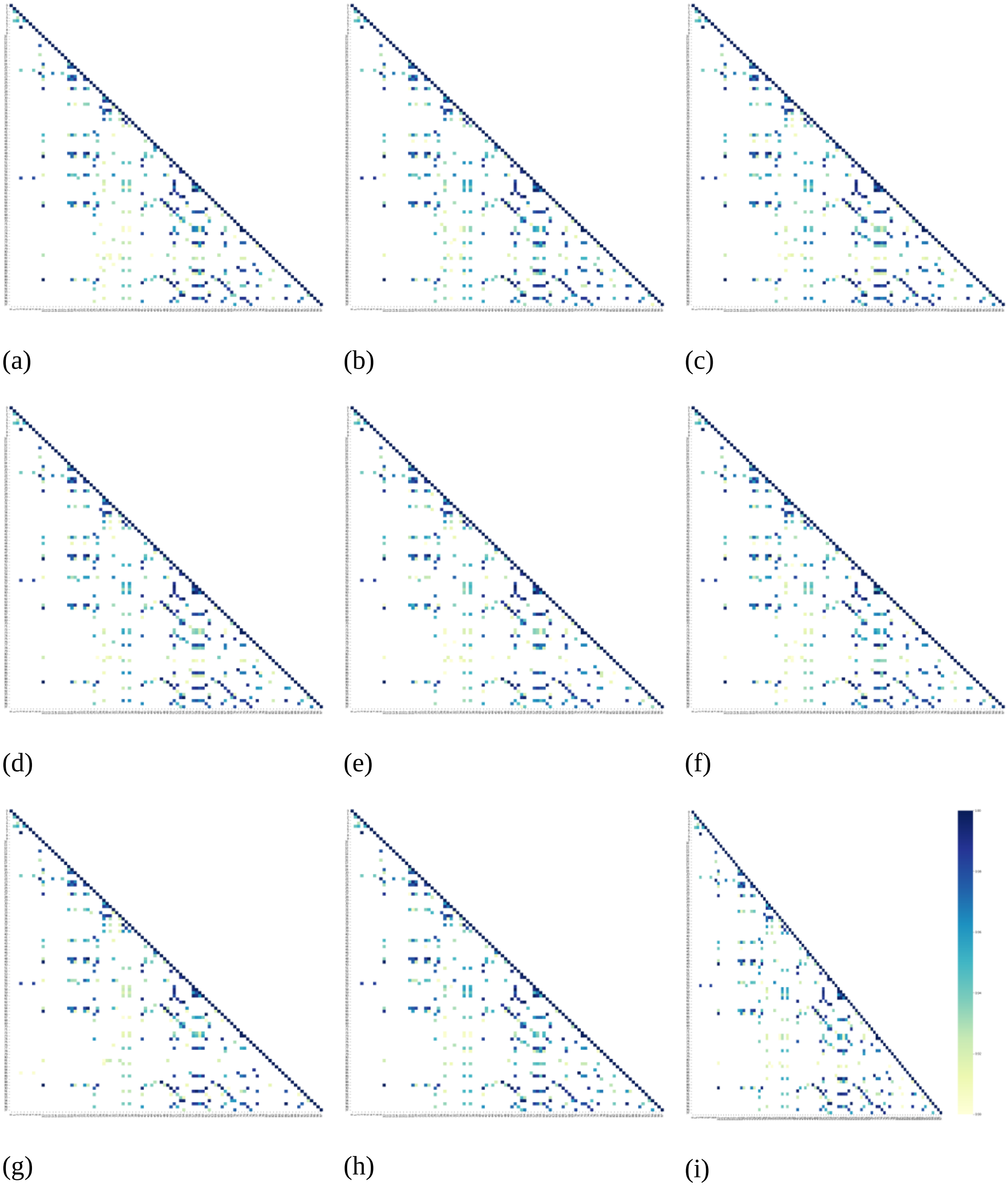

Figure S10. Examples of heatmaps of significant SCCs with absolute value greater than 0.9 for T2 maps, at fixed resampling voxel size of 2.1 mm and discretization bin width of 0.49 ms (S10a), 0.50 ms (S10b), 0.51 ms (S10c), 0.52 ms (S10d), 0.53 ms (S10e), 0.54 ms (S10f), 0.55 ms (S10g), 0.56 ms (S10h), and 0.57 ms (S10i). Numerical labels and related radiomic features are reported in Table S1.

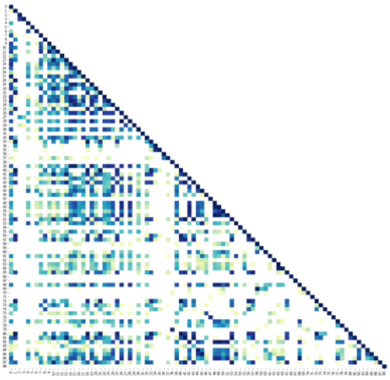

(a)

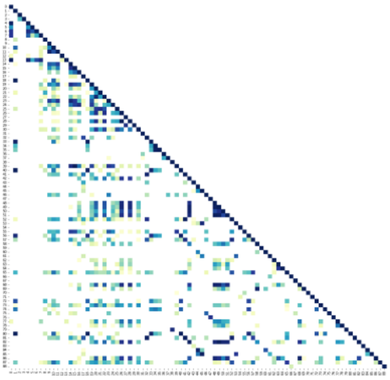

(b)

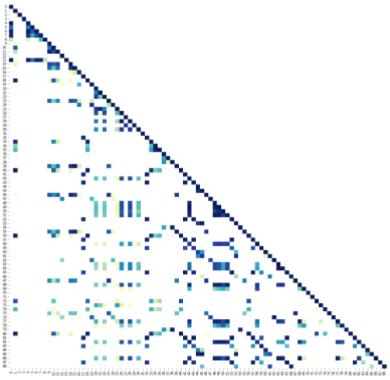

(c)

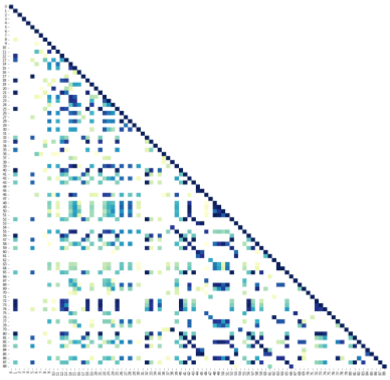

(d)

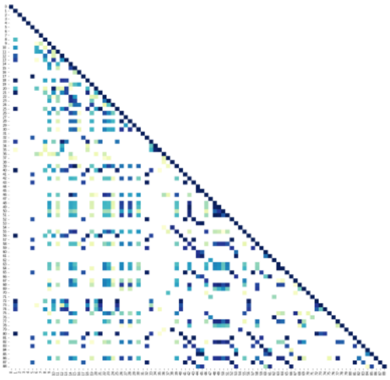

(e)

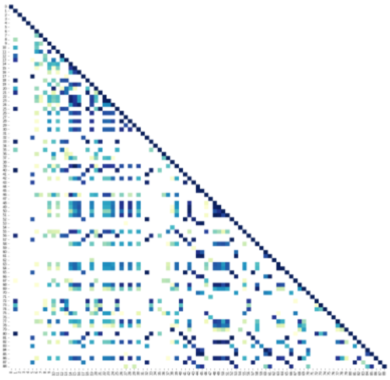

(f)

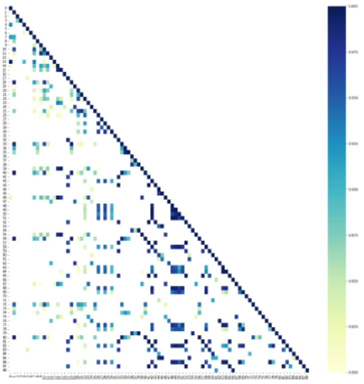

(g)

Figure S11. Heatmaps of significant PCCs with absolute value greater than 0.8 for T2 maps, at fixed resampling voxel size of 2.1 mm and discretization bin width of 6 ms, with varying filtering: gradient (S11a), square (S11b), square-root (S11c), wavelet-LH (S11d), wavelet-HL (S11e), wavelet-HH (S11f), and wavelet-LL (S11g). HH: horizontal and vertical high-pass filters, HL: horizontal high-pass filter and vertical low-pass filter, LH: horizontal low-pass filter and vertical high-pass filter, LL: horizontal and vertical low-pass filters. Numerical labels and related radiomic features are reported in Table S1.

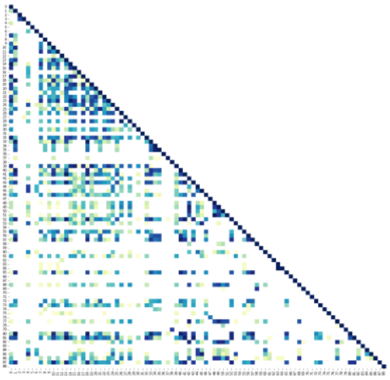

(a)

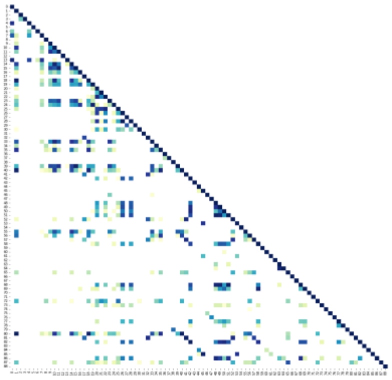

(b)

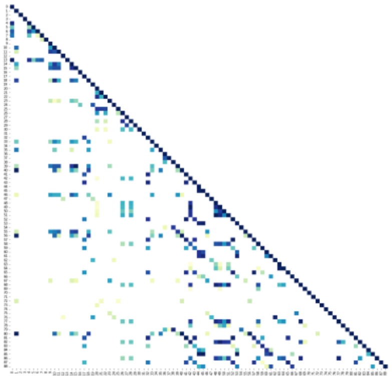

(c)

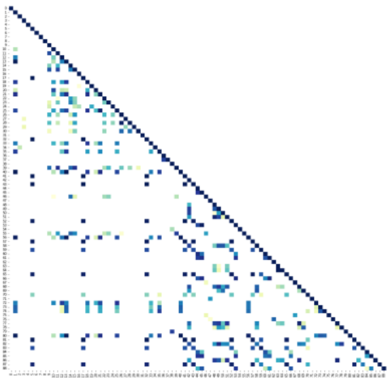

(d)

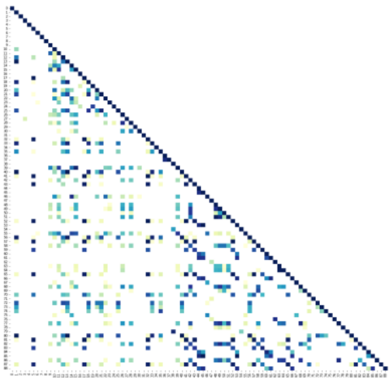

(e)

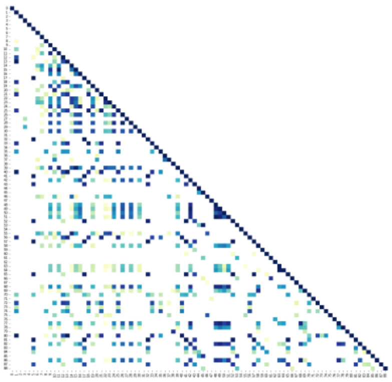

(f)

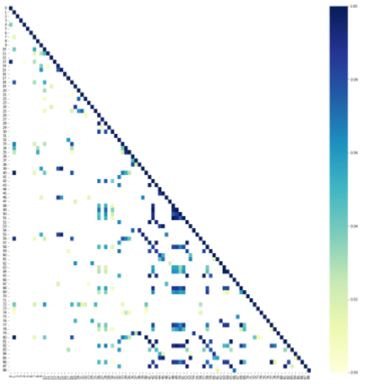

(g)

Figure S12. Heatmaps of significant SCCs with absolute value greater than 0.9 for T2 maps, at fixed resampling voxel size of 2.1 mm and discretization bin width of 6 ms, with varying filtering: gradient (S12a), square (S12b), square-root (S12c), wavelet-LH (S12d), wavelet-HL (S12e), wavelet-HH (S12f), and wavelet-LL (S12g). HH: horizontal and vertical high-pass filters, HL: horizontal high-pass filter and vertical low-pass filter, LH: horizontal low-pass filter and vertical high-pass filter, LL: horizontal and vertical low-pass filters. Numerical labels and related radiomic features are reported in Table S1.
